# Emerging strategies for combating *Fusobacterium nucleatum* in colorectal cancer treatment: Systematic review, improvements and future challenges

**DOI:** 10.1002/EXP.20230092

**Published:** 2023-12-22

**Authors:** Hongyu Liu, Yunjian Yu, Alideertu Dong, Mahmoud Elsabahy, Ying‐Wei Yang, Hui Gao

**Affiliations:** ^1^ State Key Laboratory of Separation Membranes and Membrane Processes School of Materials Science and Engineering Tiangong University Tianjin P. R. China; ^2^ College of Chemistry and Chemical Engineering Inner Mongolia University Hohhot P. R. China; ^3^ Department of Pharmaceutics Faculty of Pharmacy Assiut University Assiut Egypt; ^4^ International Joint Research Laboratory of Nano‐Micro Architecture Chemistry College of Chemistry Jilin University Changchun P. R. China

**Keywords:** antibacterial, anti‐biofilm, colorectal cancer, drug resistance, Fusobacterium nucleatum

## Abstract

Colorectal cancer (CRC) is generally characterized by a high prevalence of *Fusobacterium nucleatum* (*F. nucleatum*), a spindle‐shaped, Gram‐negative anaerobe pathogen derived from the oral cavity. This tumor‐resident microorganism has been closely correlated with the occurrence, progression, chemoresistance and immunosuppressive microenvironment of CRC. Furthermore, *F. nucleatum* can specifically colonize CRC tissues through adhesion on its surface, forming biofilms that are highly resistant to commonly used antibiotics. Accordingly, it is crucial to develop efficacious non‐antibiotic approaches to eradicate *F. nucleatum* and its biofilms for CRC treatment. In recent years, various antimicrobial strategies, such as natural extracts, inorganic chemicals, organic chemicals, polymers, inorganic‐organic hybrid materials, bacteriophages, probiotics, and vaccines, have been proposed to combat *F. nucleatum* and *F. nucleatum* biofilms. This review summarizes the latest advancements in anti‐*F. nucleatum* research, elucidates the antimicrobial mechanisms employed by these systems, and discusses the benefits and drawbacks of each antimicrobial technology. Additionally, this review also provides an outlook on the antimicrobial specificity, potential clinical implications, challenges, and future improvements of these antimicrobial strategies in the treatment of CRC.

## Graphical abstract

This review summarizes strategies to combat *F. nucleatum* and *F. nucleatum* biofilms for enhanced treatment of CRC, including natural extracts, inorganic chemicals, organic chemicals, polymers, inorganic‐organic hybrid materials, bacteriophages, probiotics, and vaccines. Additionally, the review highlights recent advances in anti‐*F. nucleatum* research, elucidates the antimicrobial mechanisms of the proposed anti‐*F. nucleatum* systems, and discusses the benefits and drawbacks of these antimicrobial technologies.

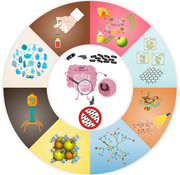

## 1 INTRODUCTION

Colorectal cancer (CRC) is responsible for more than 1.85 million cases yearly and is estimated to cause 850,000 deaths annually.^[^ ^1^ ^]^ It harbors the second‐highest global mortality rate and the third‐highest incidence rate among malignancies.^[^ ^2^ ^]^ According to a recent study, there will be over 3.2 million instances of CRC worldwide in 2040, with China and the United States leading the way in terms of the prevalence of cases during the next 20 years.^[^ ^3^ ^]^ In recent years, it has been now understood that the colon‐specific gut microbiota plays a critical role in CRC. There is proof that the abundance of *Fusobacterium nucleatum* in tumor tissues and stool specimens from CRC patients is significantly higher than in healthy controls.^[^ ^4^, ^5^, ^6^ ^]^ *F. nucleatum* is a spindle‐shaped, non‐spore‐producing Gram‐negative anaerobe bacterium and is one of the species that inhabit the human oral cavity most frequently.^[^ ^7^ ^]^ Due to its high abundance and capacity to form symbiotic relationships with other bacterial strains within the oral cavity, *F. nucleatum* serves as a crucial constituent of periodontal plaque.^[^ ^8^ ^]^ Besides, *F. nucleatum* has been discovered to profoundly affect the tumorigenesis and evolution of CRC when it enters the gut.^[^ ^9^ ^]^ The genomic DNA of *F. nucleatum* isolated from human oral and CRC tissues demonstrates the strong evolutionary link between the oral cavity and the corresponding tumor isolates.^[^ ^10^, ^11^, ^12^ ^]^ *F. nucleatum* propels CRC development through a variety of pathways. On one hand, fibroblast activation protein 2 (Fap2) and *Fusobacterium* adhesin A (FadA) play a significant role in promoting the adherence of *F. nucleatum* to intestinal epithelial cells.^[^ ^10^, ^13^ ^]^ By attaching to tumor‐expressed Gal‐GalNAc, Fap2 promotes *F. nucleatum* colorectal adenocarcinoma enrichment.^[^ ^10^ ^]^ Meanwhile, FadA adhesin from *F. nucleatum* stimulates carcinogenesis by upregulating Annexin A1 expression through E‐cadherin.^[^ ^14^ ^]^ On the other hand, *F. nucleatum* increases the risk of intestinal tumorigenesis in *Apc* ^Min/+^ mice through the Toll‐like receptor 4 (TLR4)/phosphorylated‐PAK1 (p‐PAK1)/phosphorylated‐β‐catenin S675 (p‐β‐catenin S675) cascade.^[^ ^15^, ^16^ ^]^ It has also been suggested that *F. nucleatum* manipulated colorectal cancer stem‐like cells (CCSCs) to promote CRC progression and triggered the self‐renewal of CCSCs via modulation of cellular lipid accumulation.^[^ ^17^ ^]^ *F. nucleatum* downregulates antitumor T cell‐mediated adaptive immunity.^[^ ^18^ ^]^ Some findings support the idea that *F. nucleatum* may be associated with pro‐tumoral immune responses in microsatellite instability (MSI)‐high CRC.^[^ ^19^ ^]^ The mechanism of tumor immune escape involves the Fap2 protein of *F. nucleatum*, which uses T‐cell immunoglobulin and the ITIM domain (TIGIT) to block immune cell activity.^[^ ^20^, ^21^ ^]^ Furthermore, *F. nucleatum* recruits tumor‐infiltrating immune cells, resulting in a pro‐inflammatory microenvironment that promotes the progression of colorectal neoplasia.^[^ ^9^ ^]^ Additionally, a work indicates an immunosuppressive effect of *F. nucleatum* by promoting M2 polarization of macrophages through a TLR4‐dependent mechanism.^[^ ^16^, ^22^ ^]^ During the drug treatment period, *F. nucleatum* promotes chemoresistance to the chemotherapy drug 5‐fluorouracil by upregulation of Baculoviral IAP repeat‐containing protein 3 (BIRC3) expression in CRC.^[^ ^23^ ^]^ Similarly, *F. nucleatum* orchestrates the TLR4‐MYD88, miR18a* and miR4802, and unc‐51‐like kinase 1 (ULK1)/autophagy related 7 (ATG7) autophagy networks to biologically control CRC chemoresistance.^[^ ^24^ ^]^ *F. nucleatum* promotes CRC cell metastasis by activating autophagy signaling, inhibiting methyltransferase‐like 3 (METTL3), initiating the nuclear factor kappa‐light‐chain‐enhancer of activated B cells (NF‐κB), inducing long non‐coding RNA endogenous retroviral‐associated adenocarcinoma RNA (EVADR).^[^ ^25^, ^26^, ^27^, ^28^, ^29^, ^30^, ^31^ ^]^ According to these results, the presence of intratumor *F. nucleatum* in tissues represents a biomarker for CRC. Elimination of *F. nucleatum* has become the primary prerequisite for treating CRC.

Besides, *F. nucleatum* expresses several adhesins, such as RadD, Aid1, and FomA, on its surface, facilitating bacterial co‐aggregation and biofilm formation.^[^ ^32^, ^33^, ^34^ ^]^ *F. nucleatum*‐dominant biofilms are frequently found in CRC, particularly in cases of right‐sided colon cancer.^[^ ^35^, ^36^, ^37^ ^]^ As reported by a recent study, bacterial biofilms were more frequently found in proximal CRC (89%) than distal CRC (12%).^[^ ^38^ ^]^ Following metabolomics research it was revealed that bacterial biofilms in the colonic mucosa have significant pro‐carcinogenic potential. This outcome is consistent with previous findings of bacterial biofilms containing mucus‐invasive species and an abundance of *F. nucleatum* in the proximal CRC.^[^ ^24^ ^]^ Meanwhile, a proteomic method utilizing mass spectrometry and two‐dimensional gel electrophoresis (2D‐PAGE) was employed to evaluate the differences in protein expression of *F. nucleatum* when growing in biofilms as opposed to a planktonic condition.^[^ ^39^ ^]^ The varying levels of enzyme expression responsible for microbial metabolite production during biofilm formation by *F. nucleatum* indicate the potential pathogenicity of the biofilms in both periodontal diseases and human CRC. These findings imply that the metabolic process involved in the production of *F. nucleatum* biofilms is critical to the pathogenicity of this organism in diverse body regions, such as the oral cavity or human CRC. Therefore, it is crucial to combat the harmful effects of *F. nucleatum* and prevent biofilm formation to effectively treat CRC.

Antibiotics are a type of antimicrobial substance that is effective against bacteria and is frequently utilized to prevent and treat bacterial infections.^[^ ^40^ ^]^ However, this can have a far‐reaching and immediate impact on the gut microbiota, with changes in community composition and a decline in diversity occurring within 3∼4 days after taking the medication.^[^ ^41^ ^]^ In addition, the improper and repeated use of antibiotics might cause bacteria to evolve, resulting in the development of resistance.^[^ ^42^, ^43^ ^]^ Due to the drawbacks mentioned above, this review does not cover traditional antibiotics or their delivery systems for combating *F. nucleatum*.

**SCHEME 1:**
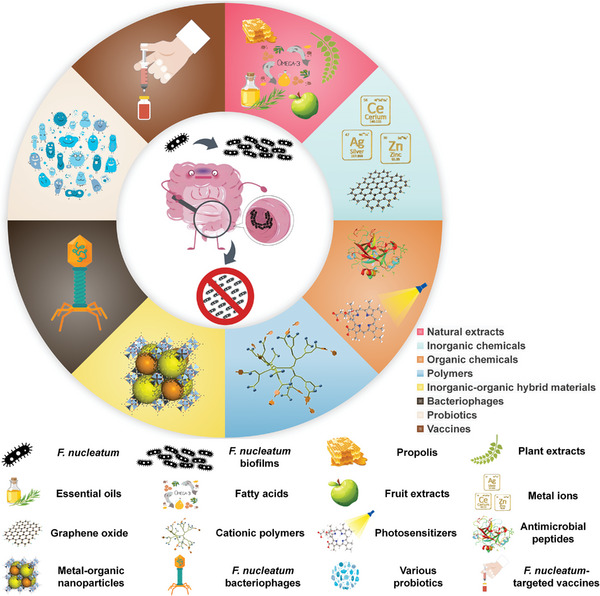
Cartoon depiction showing the proposed strategies based on natural extracts, inorganic chemicals, organic chemicals, polymers, inorganic–organic hybrid materials, bacteriophages, probiotics, and vaccines for combating *F. nucleatum* and *F. nucleatum* biofilms.

In this review, various strategies are discussed, including the utilization of natural extracts, inorganic chemicals, organic chemicals, polymers, inorganic‐organic hybrid materials, bacteriophages, probiotics, and vaccines, to specifically inhibit *F. nucleatum* and *F. nucleatum* biofilms (Scheme 1). Diverse well‐established antibacterial technologies, each with distinct advantages, offer enormous practical application potential for combating *F. nucleatum*. In the first part, we will discuss the emerging anti‐*F. nucleatum* bioactive chemicals of natural extracts derived from plant and fruit resources or animal fats.^[^ ^44^, ^45^ ^]^ Antibacterial inorganic chemicals against *F. nucleatum* will be covered in the second part, which includes metal ions and graphene oxide (GO).^[^ ^46^, ^47^ ^]^ The third part comprises organic chemicals, including photosensitizers (PSs), antimicrobial peptides (AMPs), and other compounds.^[^ ^48^, ^49^, ^50^, ^51^ ^]^ The fourth part discusses the inhibitory effects of polymers against *F. nucleatum* and presents examples of using antibacterial polymers to suppress drug‐resistant CRC.^[^ ^52^, ^53^, ^54^, ^55^, ^56^ ^]^ In the following section, inorganic‐organic hybrid materials for *F. nucleatum* killing will be presented. Some dual‐combination antibacterial strategies will open up new ideas for *F. nucleatum* inhibition.^[^ ^57^, ^58^ ^]^ Bacteriophages with specific bacterial targets appear to be a promising option for combating *F. nucleatum*.^[^ ^59^ ^]^ Bacteriophages’ morphology, genomics, and functional characteristics will be described in the sixth part.^[^ ^60^ ^]^ The seventh part will go into the topic of probiotics as a potential substitute for conventional antibiotics. When ingested in sufficient quantities, probiotics confer health benefits to the host organism.^[^ ^61^, ^62^ ^]^ Certain strains have demonstrated in vitro antagonistic activity against *F. nucleatum*.^[^ ^63^, ^64^ ^]^ Vaccines are another method for reducing the overall load of resistant or susceptible microorganisms while simultaneously lowering antibiotic usage.^[^ ^65^ ^]^ In part eight of the review, *F. nucleatum*‐targeted vaccines that elicit an immune response will be introduced.^[^ ^66^ ^]^

To provide a comprehensive and up‐to‐date review of the anti‐*F. nucleatum* field, this review will cover the antimicrobial mechanisms of various antibacterial materials and their potential in combating pathogenic *F. nucleatum*. Furthermore, the review will discuss the prospects and obstacles of developing these strategies to inspire scientists to generate new ideas for controlling *F. nucleatum* and *F. nucleatum* biofilms in CRC and advancing this research field in the future.

## 2 NATURAL EXTRACTS

### 2.1 Propolis and plant extracts

Medicinal chemists are inspired by natural products to develop new antibacterial materials and have extracted robust and effective antibacterial agents based on natural products against *F. nucleatum* (Table 1). A possible source of bioactive substances with pharmacological characteristics, such as antibacterial, anti‐inflammatory, and antiviral properties, has been identified in propolis.^[^ ^67^, ^68^, ^69^ ^]^ It has been discovered that flavonoids in propolis extracts exert antibacterial effects on *F. nucleatum* while circumventing the development of *F. nucleatum* biofilms in vitro.^[^ ^70^, ^71^ ^]^ The use of medicinal plants around the world makes a significant contribution to primary healthcare.

**TABLE 1:** Natural products against *F. nucleatum* and *F. nucleatum* biofilms.

Nature product	Active antibacterial compound	Antibacterial mechanism	Refs.
Oxapampa propolis	Flavonoids	Curcumin inhibits DNA replication, modifies gene expression, destroys cell membranes, and decreases microbial motility.^[^ ^95^, ^96^ ^]^	[70]
Kopaonik propolis	Flavonoids		[71]
CCHs	Curcumin, cinnamaldehyde		[85]
Turmeric rhizome	Curcuminoid	Flavonoids limit nucleic acid production, energy metabolism, and membrane permeability.^[^ ^97^ ^]^	[81]
Turmeric extract	Curcuminoid		[82]
Curcuma xanthorrhiza Roxb.	Curcuminoid		[74]
Isabgol	Acid derivatives	Acid derivatives enhance the permeability of cell membranes, causing the barrier function to be disrupted and a modest amount of nucleotide leakage.^[^ ^104^, ^105^ ^]^	[86]
Aegiceras corniculatum	Alkaloid, saponin, flavonoids, terpene, tannin, quinone		[87]
Thesium chinense	Acid derivatives		[88]
Ilex hainanensis Merr.	Terpene	Alkaloids inhibit bacterial nucleic acid and protein synthesis and modify bacterial cell membrane permeability.^[^ ^99^ ^]^	[89]
Bauhinia forficata tincture	Phenol, flavonoids, acid derivatives		[90]
Berberis goudotii	Tannin, polyphenol		[76]
Stem‐leaf American ginseng	Saponin	Terpenes impede the peptidation of the expanding peptidoglycan chain and cell wall production enzymes.^[^ ^100^ ^]^	[91]
Caesalpinia ferrea Mart. (Jucá)	–		[83]
Cinnamon oil	Cinnamaldehyde		[84]
Cissus quadrangularis, Aloe castellorum, Psiadia punctulata, Aloe pseudorubroviolacea, Barbeya oleoides, Teucrium yemense	Phenol, flavonoids, alkaloid, tannin, saponin,	Tannin penetrates the bacterial cell wall up to the interior membrane and affects the metabolism of the cell.^[^ ^101^ ^]^	[92]
Salvia officinalis L.	–		[75]
Malacomeles denticulata	Flavonoids, saponin		[93]
Red pomegranate	Alkaloid, flavonoid, Polyphenol	Quinones damage cell wall constituents including cell wall polypeptides, and membrane‐bound enzymes.^[^ ^102^ ^]^	[77]
Young apple polyphenols	Polyphenol		[94]
Bilberry	–		[78]
Cranberry juice	Polyphenol	Polyphenols damage the bacterial membrane, with changes in permeability, polarization, and interruption in efflux activity.^[^ ^103^ ^]^	[79]
Cranberry extracts	Polyphenol		[80]
Quinoa	Saponin		[95]
Labrador tea and peppermint and winter savory	Terpene		[111]
L. Scoparium	Terpene	Cinnamaldehydes contacting the cell membrane cause a fast blockage of energy metabolism and a leak of phosphate ions.^[^ ^106^, ^107^ ^]^	[112]
Psidium cattleianum leaves	Terpene		[113]
Hinokitiol	Terpene	Saponin causes perforation and rupture of the bacterial cell membrane.^[^ ^98^ ^]^	[114]
Perillyl alcohol	Terpene		[115]

**FIGURE 1:**
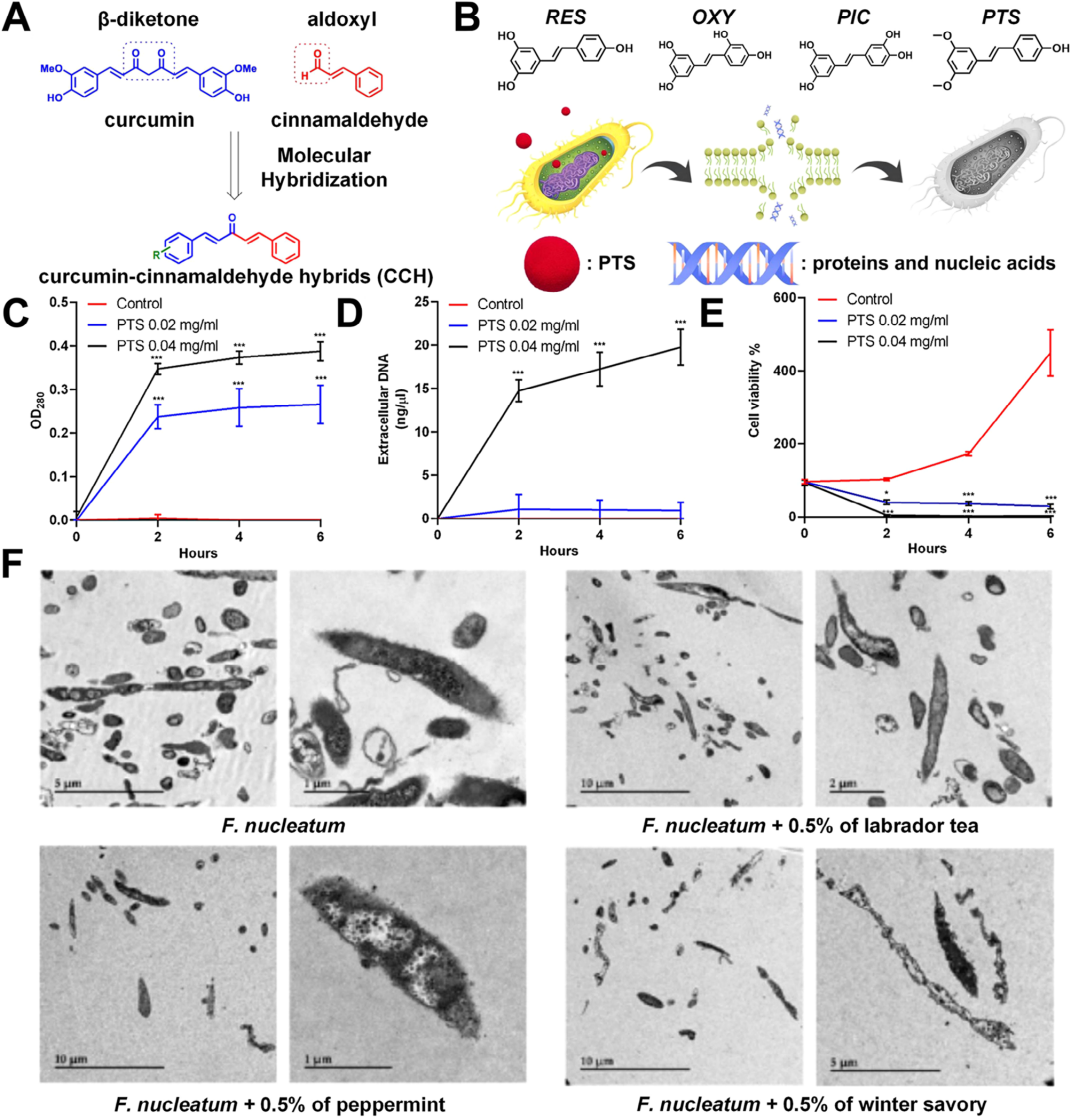
Design of CCHs: (A) Synthetic route of CCHs. Reproduced with permission.^[^ ^85^ ^]^ Copyright 2021, Taylor & Francis. Structure of PTS and its anti‐*F. nucleatum* properties: B) Chemical structures of RES, PIC, OXY, and PTS. PTS triggered leakage of bacterial proteins and nucleic acids. The content of extracellular (C) proteins and (D) nucleic acid in *F. nucleatum* after treatment with PTS for 2, 4, and 6 h. (E) Cell viability of *F. nucleatum* after treatment with PTS. Reproduced with permission.^[^ ^108^ ^]^ Copyright 2020, Springer Nature. Destructive effects of three EOs on *F. nucleatum*: (F) TEM images of *F. nucleatum* after different treatments for 1 h. Reproduced with permission.^[^ ^111^ ^]^ Copyright 2020, MDPI.

So far, numerous novel antibacterial substances have been discovered in a variety of plants.^[^ ^72^ ^]^ These new compounds provide alternatives for combating multidrug resistance in microorganisms.^[^ ^73^ ^]^ As a result, screening plant extracts is an advisable approach to seeking active antibacterial compounds against *F. nucleatum*.^[^ ^74^, ^75^, ^76^, ^77^, ^78^, ^79^ ^]^ Many components found in herbaceous plants and fruits, such as polyphenols, flavonoids, terpenes, alkaloids, steroids, quinones, saponins, and acidic compounds, have been shown to inhibit *F. nucleatum*.^[^ ^80^, ^81^, ^82^, ^83^, ^84^ ^]^ Curcumin is a plant polyphenol extracted from turmeric that was hybridized with cinnamaldehyde by Duque et al. to produce a new small‐molecule antimicrobial agent. Curcumin‐cinnamaldehyde hybrids (CCHs) have a minimum inhibitory concentration (MIC) and a minimum bactericidal concentration (MBC) range of 9 to 625 μg mL^−1^ against *F. nucleatum*.^[^ ^85^ ^]^ These findings demonstrated that molecular hybridization could successfully create a new antimicrobial compound, CCH 7 (MIC = 9 μg mL^−1^, MBC = 19 μg mL^−1^), with a thirty‐fold increase in antibacterial activity compared to the initial compounds of cinnamaldehyde and curcumin (Figure 1A). However, CCH 7 did not affect *F. nucleatum* biofilms or mixed bacterial biofilms. Tan et al. aimed to compare the antibacterial activity of natural polyphenol resveratrol (RES) and its analogs (pterostilbene (PTS), oxyresveratrol (OXY), and piceatannol (PIC)), utilizing 2‐hydroxypropyl‐β‐cyclodextrin (Hp‐β‐CD) as a solubilizer (Figure 1B).^[^ ^108^ ^]^ The results implied that when complexed with Hp‐β‐CD, PTS owned the best antibacterial effect on *F. nucleatum*, while the MIC and MBC values were measured to be 0.02 mg mL^−1^ and 0.04 mg mL^−1^, respectively. PTS complexed with HP‐β‐CD demonstrated significant antibacterial activities against *F. nucleatum* with at least a sixty‐fold increase over RES, OXY, and PIC. In addition, 0.02 mg mL^−1^ of PTS caused substantial leakage of bacterial proteins into the extracellular environment (Figure 1C), and further triggered the leakage of both bacterial proteins and nucleic acids when the concentration was 0.04 mg mL^−1^ (Figure 1C,D). This phenomenon was most obvious within the first 2 h of *F. nucleatum* treatment with PTS. Cellular content leakage was detected in tandem with a decline in bacterial viability (Figure 1E). Since PTS was more effective at killing *F. nucleatum* than RES and other similar compounds, PTS complexed with Hp‐β‐CD presented the most significant potential for eliminating CRC‐associated *F. nucleatum* infection.

Essential oils (EOs) are colorless liquids comprising predominantly aromatic and naturally occurring volatile organic components found in plants’ seeds, flowers, peel, stem, bark, and whole plants.^[^ ^109^ ^]^ EOs deploy special medicinal advantages, such as antibacterial and antifungal properties, due to their role as secondary plant metabolites essential for plant survival processes.^[^ ^110^ ^]^ The main anti‐*F. nucleatum* components in EOs are monoterpenes: sabinene, menthone, and carvacrol; γ‐terpinene and sesquiterpenes: α‐ and β‐selinene, germacrene, and viridiflorol (Table 1).^[^ ^111^, ^112^, ^113^, ^114^, ^115^ ^]^ Grenier et al. investigated the effects of EOs from three plants: Labrador tea (Rhododendron groenlandicum Kron & Judd), peppermint (Mentha x piperita L.), and winter savory (Satureja montana L.), on inhibiting *F. nucleatum* growth and biofilm formation.^[^ ^111^ ^]^ Their research reported the chemical composition of the three EOs used in the study in terms of volatile components. *F. nucleatum* without treatment was observed by transmission electron microscopy (TEM) to reveal a normal bacillary morphology and electron opacity. *F. nucleatum*, on the other hand, underwent significant cell alterations treatment with different EOs (Figure 1F).

Combining natural product extracts with polysaccharide carriers to obtain nanomaterials with improved antimicrobial properties also presents new ideas for the antimicrobial application of natural products. Naguib et al. created nano defensins as antimicrobial agents.^[^ ^116^ ^]^ Fenugreek seeds were used to extract and purify defensin, which was then immobilized on nano chitosan to create nano defensin. The MIC of nano defensins (10.6 µg mL^−1^) against *F. nucleatum* was found to be much lower than that of free defensins (66 µg mL^−1^) and nano‐chitosan (339.4 µg mL^−1^). The potential mechanism of nano defensin against *F. nucleatum* was assigned to the destruction of bacterial membranes and their genetic substances. Simultaneously, the stability and activity of defensin were boosted by immobilization, thereby protecting it from bacterial enzyme hydrolysis.

Natural extracts are naturally obtained and, when used in moderation, do not cause significant harm to the human body. They can be extracted into the desired molecules, eliminating the need for complex synthetic steps. Compared with synthetic chemicals, natural products are more architecturally complex, permitting greater specificity toward biological targets, and exhibit a higher possibility of serving as substrates for transporter systems that promote their transportation into bacterial cells.^[^ ^117^, ^118^, ^119^, ^120^ ^]^ Meanwhile, natural extracts have been reported to not only undergo a metabolic transformation in the presence of intestinal flora but also balance the ecological structure of intestinal flora.^[^ ^121^ ^]^ A previous study that investigated the impact of dietary supplementation with curcumin on the human intestinal environment indicated the beneficial role of curcumin on the proliferation and growth of intestinal probiotics.^[^ ^122^ ^]^

However, antibacterial natural extracts are vulnerable to fluctuations in composition due to seasonal changes and environmental unpredictability, resulting in the loss of antibacterial properties.^[^ ^123^ ^]^ Up to now, natural extracts were utilized to inhibit *F. nucleatum* inhibited by only in the oral cavity and were confined to in vitro investigations. There were few examples that controlled *F. nucleatum* at the CRC site by using natural extracts, probably due to their poor oral bioavailability.^[^ ^124^ ^]^ The introduction of nano‐carriers may improve the bioavailability of natural extracts for being applied in vivo. For example, xylan, a natural polysaccharide extracted from corn cobs, was used as a drug delivery tool, especially in the colon.^[^ ^125^ ^]^ Due to its timely retention in the physiological milieu of the stomach and small intestine, xylan was only degraded by anaerobic microorganisms in the colon. A separate study found that curcumin loaded on the nanolipid exhibited a sustained release pattern compared to that of curcumin solution.^[^ ^126^ ^]^ The employment of a suitable carrier not only endow antibacterial natural extracts with specific enrichment in the colon but attach with long‐lasting drug delivery performance.

### 2.2 Oil and fat extracts

Fatty acids (FAs) are organic compounds with long, straight, branched, saturated, or unsaturated aliphatic chains and carboxylic acids, which are found in oils and fats from plants and animals.^[^ ^127^ ^]^ Evidence suggests that saturated and unsaturated FAs can inhibit bacterial adhesion and subsequent biofilm formation.^[^ ^128^, ^129^ ^]^ In the antibacterial aspect, common antibacterial mechanisms of FAs typically work by preventing DNA/RNA replication, halting cell wall biosynthesis, inhibiting protein synthesis, provoking cytoplasmic membrane disruption, and restraining metabolic routes.^[^ ^130^ ^]^ In this section, the effect of several free FAs and straight‐chain FAs that are covalently linked to nanoparticles (NPs) on the growth of *F. nucleatum* will be discussed.

Sanz et al. used an in vitro biofilm model composed of multiple species, including *F. nucleatum*, to evaluate the antibacterial efficacy of two omega‐3 FAs.^[^ ^131^ ^]^ The anti‐biofilm activity of eicosapentaenoic acid (EPA) and docosahexaenoic acid (DHA) showed a decrease of three orders of magnitude compared to the control group. As previously stated, polyunsaturated FAs can enter the plasma membrane, weakening its integrity and making it more permeable. Sette‐de‐Souza et al. found that *F. nucleatum* exhibited more EPA and DHA targets than the other pathogens studied in a computer‐based analysis of protein‐acid interactions, protein characterization, and molecular docking.^[^ ^132^ ^]^ EPA, for example, interacts with the tolC in *F. nucleatum*. Bacteria decrease acid pH resistance by occluding the tolC canal or interfering with the toxic molecule efflux, which might lead to growth abnormalities and metabolic shutdown. By inactivating this protein, bacteria become susceptible to various antimicrobial agents. As a type of FAs, EPA demonstrates potential antibacterial ability against *F. nucleatum*.

**FIGURE 2:**
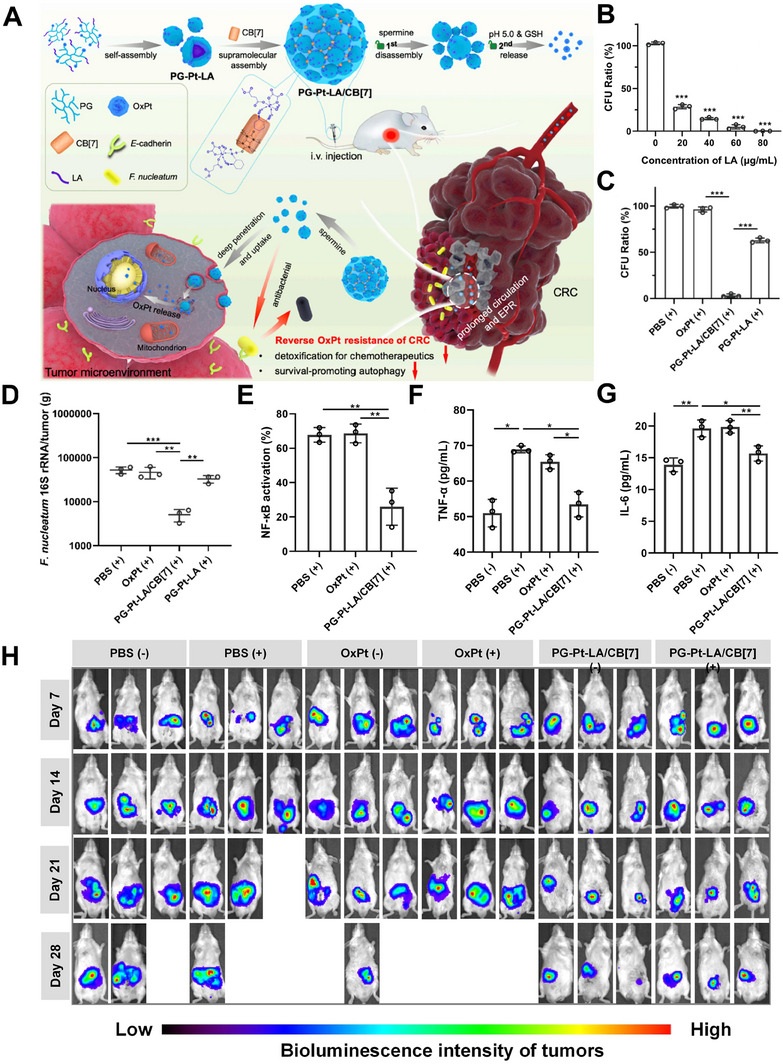
LA‐loaded supramolecular nanosystem for addressing drug‐resistance CRC based on the inhibition of *F. nucleatum*: (A) Schematic diagram of supramolecular nanomedicine for enhanced systemic chemotherapy against drug‐resistant CRC. (B) The spread plate and quantification of intratumor *F. nucleatum* exposure to LA at various concentrations. (C) The quantification of intratumor *F. nucleatum* after various treatments. (D) Quantitative real‐time PCR detection of intratumor *F. nucleatum* 16S rRNA expression. (E–G) Expression of NF‐κB, TNF‐α, and IL‐6 in tumor tissues infected by *F. nucleatum* measured by enzyme‐linked immunosorbent assay (ELISA), where (+) and (−) indicates incubation microenvironments in the presence or absence of *F. nucleatum*, respectively. (H) In vivo bioluminescence images of mice following treatment with PBS (−), PBS (+), OxPt (−), OxPt (+), PG‐Pt‐LA/CB[7] (−), and PG‐Pt‐LA/CB[7] (+) (where (+) and (−) referred to incubation in the presence or absence of *F. nucleatum*, respectively). Reproduced with permission.^[^ ^135^ ^]^ Copyright 2022, Elsevier.

Lauric acid (LA), a natural FA, exhibited a selective antibacterial impact on *F. nucleatum*.^[^ ^133^ ^]^ Our group recently fabricated a new dendrimer‐based nanomaterial, polyamidoamine (PAMAM)‐platinum (Pt)‐LA, with anti‐CRC and anti‐*F. nucleatum* dual‐functions. PAMAM‐Pt‐LA@HA was afforded by coating PAMAM‐Pt‐LA with hyaluronic acid (HA) to afford.^[^ ^134^ ^]^ *F. nucleatum* could secrete hyaluronidase, which decomposes the coated HA of the PAMAM‐Pt‐LA@HA nano‐complex and induces the release of LA, thereby inhibiting *F. nucleatum*. Further, when HCT116 (human colorectal carcinoma cells) were infected with *F. nucleatum*, the released LA from PAMAM‐Pt‐LA@HA inhibited the proliferation of *F. nucleatum* and decreased the chemotherapy resistance, allowing oxaliplatin to better inhibit the growth of CRC cells. To resolve chemotherapy resistance caused by *F. nucleatum*, we further developed a multifaceted supramolecular nanomedicine, polyglycidyl ether (PG)‐Pt‐LA/cucurbit[7]uril (CB[7]) (Figure 2A).^[^ ^135^ ^]^ The chemotherapeutic effect of oxaliplatin is promoted by using LA to eradicate *F. nucleatum*. Pure LA demonstrated the same killing outcome against *F. nucleatum* at the same LA concentration of PG‐Pt‐LA/CB[7] (Figure 2B), demonstrating that LA was the major source of the antibacterial actions of PG‐Pt‐LA/CB[7]. When LA was added, NPs effectively inhibited the growth of *F. nucleatum* at the tumor site (Figure 2C). Quantitative real‐time polymerase chain reaction (PCR) was used to identify the expression of *F. nucleatum* 16S rRNA in the tumor, and the results showed a drastically declining abundance of *F. nucleatum* (Figure 2D). Then, the antitumor potency of PG‐Pt‐LA/CB[7] was confirmed by using an orthotopic CRC model. PG‐Pt‐LA/CB[7] displayed a remarkable inhibition effect on the tumors infected with *F. nucleatum* (referred to as (+)) (Figure 2H), suggesting that PG‐Pt‐LA/CB[7] could circumvent the chemotherapy resistance of CRC induced by intratumor *F. nucleatum*. Additionally, PG‐Pt‐LA/CB[7] could greatly decrease the expression of NF‐κB to 25.9% (Figure 2E). Meanwhile, PG‐Pt‐LA/CB[7] treatment significantly reduced the gene expression of the cytokines tumor necrosis factor (TNF‐α) and interleukin 6 (IL‐6) compared to PBS (+) and OxPt (+) (Figure 2F,G), suggesting that the nanomedicine could relieve inflammation caused by *F. nucleatum*.

Indeed, FAs are the most potent antimicrobial agents in human skin lipid samples.^[^ ^127^ ^]^ Similarly, the aforementioned FAs inhibited *F. nucleatum* effectively. Among them, LA has been successfully employed to eliminate *F. nucleatum* from the CRC site, demonstrating excellent outcomes in animal experiments when used in combination with anticancer drugs. LA, derived from natural coconut oil, is non‐toxic to living organisms in appropriate concentrations.^[^ ^136^ ^]^ Furthermore, LA reduces the hydrophobicity of bacterial cells, thereby inhibiting biofilm formation. More importantly, FAs have minimal impact on the beneficial microorganisms residing in the gastrointestinal tract. In fact, the appropriate use of probiotics and ω − 3 FAs has been shown to promote the variety of gut microbiota and mitigate low‐grade inflammation.^[^ ^137^ ^]^

However, FAs are susceptible to oxidative degradation and have limited solubility in water, making them unsuitable for effective in vivo delivery.^[^ ^138^ ^]^ Therefore, there is a need to develop efficient delivery systems that can effectively transport FAs to the colonic site for anti‐*F. nucleatum* treatment. This is an important issue that requires attention and resolution. Cansell et al. conducted a study to examine the intestinal bioavailability of FAs when carried by marine phospholipids and formulated in various supramolecular forms. Their research aimed to enhance the delivery of FAs to the intestinal site, which revealed that using marine phospholipids as a vehicle for FA delivery improved absorption efficiency.^[^ ^139^ ^]^ In future research, more sophisticated carriers need to be designed to effectively deliver FAs to the colonic site for more efficient *F. nucleatum* eradication (Table 2).

**TABLE 2:** FAs against *F. nucleatum* and *F. nucleatum* biofilms.

FAs	Nanosystem	Antibacterial mechanism	Refs.
EPA	–	EPA and DHA can enter the plasma membrane, weakening its integrity and making it more permeable, and *F. nucleatum* exhibited more EPA and DHA targets in a computer‐based analysis.^[^ ^131^, ^132^ ^]^	[131]
DHA	–		[132]
LA	PAMAM‐Pt‐LA@HA	The released LA from the nanosystem inhibited the proliferation of *F. nucleatum* and decreased the chemotherapy resistance of CRC.^[^ ^135^ ^]^	[134]
	PG‐Pt‐LA/CB[7]		[135]

## 3 INORGANIC CHEMICALS

### 3.1 Metal‐based materials

The antibacterial nature of metals has long been identified for utilization in various scenarios.^[^ ^140^ ^]^ Metal can be fabricated into nanomaterials, which provide robust antimicrobial activity at lower dosages.^[^ ^141^ ^]^ Due to their smaller size and larger surface area to volume ratio than bacteria, metal NPs can bind to the active sites of the bacteria's membrane surface as completely as possible, allowing metal ions to be released rapidly and disrupting the cell membrane potential and integrity, inhibiting *F. nucleatum* proliferation and biofilm formation.^[^ ^142^, ^143^ ^]^ An abundant amount of water from the cytosol is released because the cell barrier is broken. Bacterial cells have evolved to use proton efflux pumps and electron transport to compensate for this loss. However, the enormous demand for these ions severely harms the transmembrane systems. Overall, this ionmembrane is out of balance, which disrupts energy transfer, impairs respiration, and ultimately leads to cell death.^[^ ^144^ ^]^ In addition, metal NPs can react with sulfur‐containing proteins and phosphorus‐containing compounds in the interior of the cell. This can potentially disrupt bacterial respiratory and metabolic pathways as well as ATP production.^[^ ^145^, ^146^ ^]^ Adding the metals Zn^+^, Ag^+^, Mg^2+^, Ce^3+^/Ce^4+^, and Sr^2+^ to the various systems dramatically enhances the antibacterial and anti‐biofilm effects compared to the original systems, according to current research on *F. nucleatum* inhibition.^[^ ^147^, ^148^, ^149^, ^150^, ^151^ ^]^ We will discuss the anti‐*F. nucleatum* research metal NPs in a later section.

**FIGURE 3:**
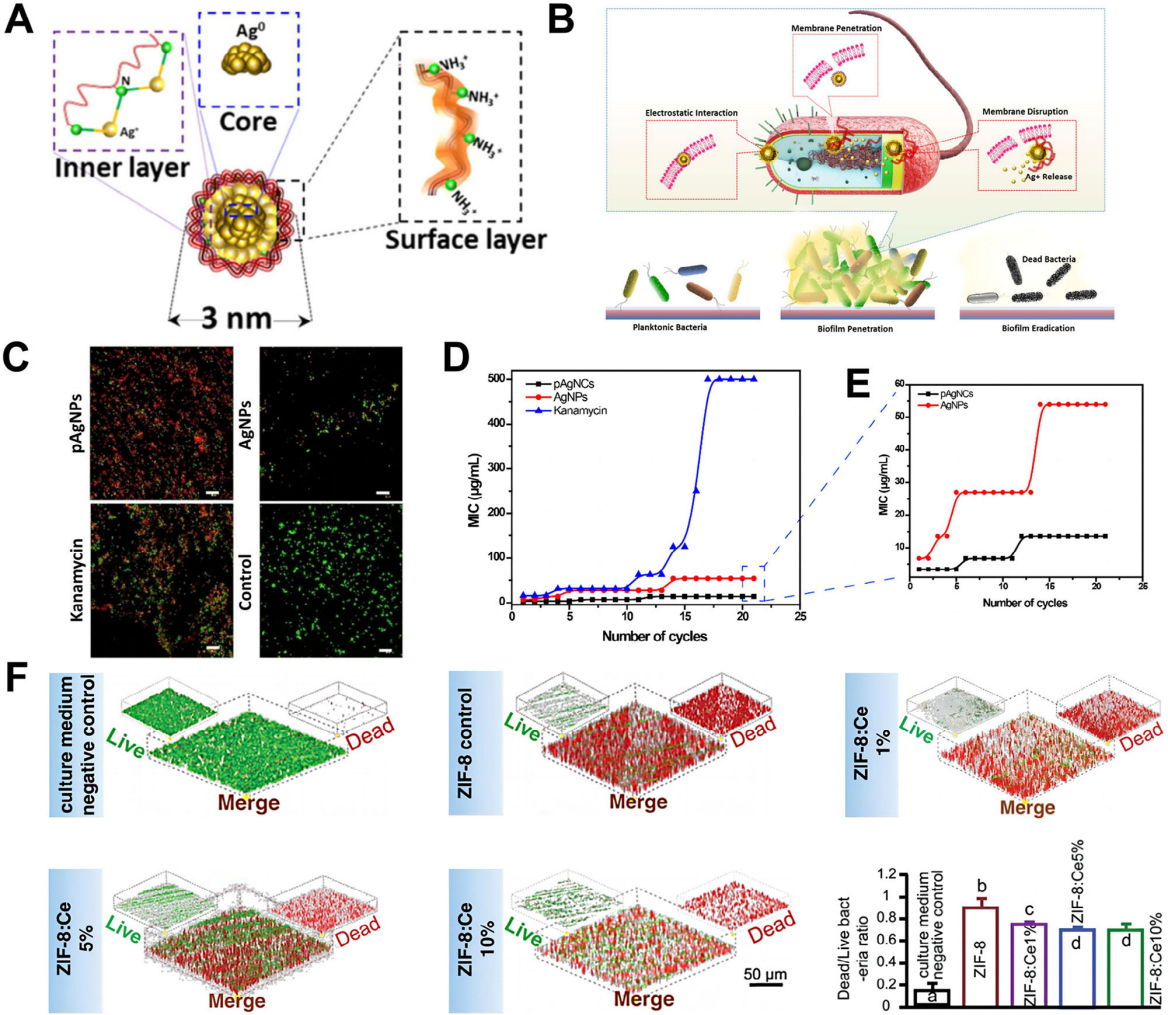
Structure, antimicrobial mechanism, and anti‐*F.nucleatum* property of pAgNCs. (A) Structure diagram of antibacterial pAgNCs. (B) Schematic illustration showing the multifaceted antibacterial mechanism of pAgNCs by penetrating and rupturing bacterial membranes. (C) Live/dead staining of *F. nucleatum* observed by CLSM. Scale bar: 10 µm. (D) Changes of MIC against *F. nucleatum* over number of cycles after treatment with pAgNCs, AgNPs and kanamycin. (E) Detailed MIC changes between pAgNCs and AgNPs. Reproduced with permission.^[^ ^152^ ^]^ Copyright 2021, American Chemical Society. The effects of ZIF‐8 or ZIF‐8:Ce NPs on inhibition of *F. nucleatum* biofilms: F) Representative CLSM‐3D live/dead photographs of *F. nucleatum* biofilms after treatment with ZIF‐8 or ZIF‐8:Ce NPs. Reproduced with permission.^[^ ^153^ ^]^ Copyright 2019, Royal Society of Chemistry.

Goswami and colleagues synthesized highly mono‐dispersed, ultrasmall (3 nm) polycationic silver nanoclusters (pAgNCs) to eliminate *F. nucleatum* (Figure 3A,B).^[^ ^152^ ^]^ According to the confocal laser scanning microscopy (CLSM) images of live/dead staining, pAgNCs significantly reduced the bacterial viability of *F. nucleatum* (Figure 3C). The MIC of kanamycin for *F. nucleatum* was already greater than 500 µg mL^−1^ after 15 cycles of drug resistance testing (Figure 3D,E), which is far too high to be clinically useful. By contrast, the MIC of pAgNCs increased slightly, reaching 13.5 µg mL^−1^ to *F. nucleatum* (up from 3.75 µg mL^−1^ originally) after 12 cycles. The ultrasmall size and positively charged surface not only increased the ability of pAgNCs to kill pathogens but also prevented microorganisms from developing resistance over time. Wang et al. developed novel ZIF‐8 NPs with 1%, 5%, and 10% Ce/(Ce + Zn) molar ratios and investigated the inhibition efficacy against *F. nucleatum* biofilms.^[^ ^153^ ^]^ It still achieved a reduction of nearly two orders of magnitude in colony‐forming units (CFUs), although 10% Ce doping in ZIF‐8 decreased the antibacterial action slightly (Figure 3F). ZIF‐8 NPs with varying Ce proportions bore on Zn content, compromising its bactericidal ability. This outcome was attributed to the lower anti‐*F. nucleatum* capacity of Ce^3+^/Ce^4+^ than Zn^2+^. Wang et al. employed hydrothermal methods to prepare different shapes of nano‐CeO_2_ (nanorod, nanocube, and nano‐octahedron). Nano‐CeO_2_ was then coated into the Ti surface, and its inhibitory effect on *F. nucleatum* biofilms was tested.^[^ ^154^ ^]^ As a result, octa‐CeO_2_ displayed the most robust inhibition against *F. nucleatum* biofilms. The octa‐CeO_2_ exhibited the highest Ce^3+^ value and biofilm removal effect due to its smallest size and the special octahedral structure that exposed more crystalline planes. Aside from the physical properties of CeO_2_, another potential antibacterial mechanism of CeO_2_ could involve the inactivation of bacterial surface proteins by the interaction between CeO_2_ and thiol groups (–SH), reducing the permeability of the cell membrane.^[^ ^155^ ^]^

In general, these metal NPs provide a fine antibacterial material that acts long against both planktonic and biofilm bacteria by causing membrane instability and protein adhesion. Metal ions, particularly Ag^+^, possess notable antibacterial activities and exhibit a wide range of effectiveness against various bacterial strains, hence conferring an advantageous attribute. Metal ions possess a reduced propensity to induce bacterial resistance and are considered to be safer when compared to organic small‐molecule antibacterial agents.

However, the consumption of metals by humans can induce alterations in the composition of the gut microbiota, therefore eliciting an indirect pro‐inflammatory response through the metabolic activities of intestinal microbes. Consequently, this might give rise to the occurrence of intestinal diseases.^[^ ^156^, ^157^ ^]^ For instance, research has demonstrated that the intake of TiO_2_ NPs is supposed to result in disruptions to the metabolic activities of intestinal microorganisms, leading to a notable elevation in the production of lipopolysaccharide (LPS).^[^ ^158^ ^]^ LPS elicits the upregulation of TLR4 expression, leading to the secretion of pro‐inflammatory cytokines and initiating the immunological response in the organism.^[^ ^159^ ^]^ In the meantime, the growth and viability of probiotic microorganisms, namely *Bifidobacterium* and *Lactobacillus*, are impacted by their exposure to metal NPs.^[^ ^160^, ^161^ ^]^

The primary requirement for addressing the proliferation of *F. nucleatum* at CRC sites, however, is to maintain a balance between metal NPs and the human body as well as the intestinal flora.^[^ ^162^ ^]^ Some probiotics have been shown to neutralize toxicity caused by metal ions in vitro and in vivo.^[^ ^163^ ^]^ The probiotic detoxification of metals is accomplished through the binding of metal ions to the bacterial cell wall, followed by accumulation inside the bacteria through cell membrane permeation.^[^ ^164^ ^]^ Therefore, probiotic preparations can be employed in synergy with metal NPs to directly reduce the accumulation of metals in the human intestine and protect the human intestine while acting as efficacious antibacterial agents (Table 3).

**TABLE 3:** Metal‐based materials against *F. nucleatum* and *F. nucleatum* biofilms.

Metal	Anti‐*F. nucleatum* system	Antibacterial mechanism	Refs.
Zn^+^	Zn‐HAp‐Ti	Zn^+^ bind to bacterial proteins and then deactivate proteins or altering the structure of cellular membranes.^[^ ^147^ ^]^	[147]
	–		[148]
Ag^+^	Multifunctional composite with NPs of Ag^+^	Ag^+^ would interact with the vital enzymes of the bacteria, leading to enzyme inactivation, further resulting in DNA in the bacteria losing its replication ability, thus causing cell death.^[^ ^152^ ^]^	[149]
	^F^G_d_Ag		[150]
	GSH‐AgNPs		[151]
	pAgNCs		[152]
Ce^3+^/Ce^4+^/CeO_2_	ZIF‐8 NPs	Ce^3+^/Ce^4+^ increases the permeability of the outer membrane of bacterial cells. CeO_2_ could involve the inactivation of bacterial surface proteins by the interaction between CeO_2_ and ‐SH.^[^ ^155^ ^]^	[153]
	nano‐CeO_2_		[154]

*Abbrevistions*: HAp, hydroxyapatite; ^F^G_d_, 2′‐deoxy‐2′‐fluoroguanosine; GSH‐AgNPs, glutathione‐stabilized silver NPs.

### 3.2 GO

GO is an oxidized version of graphene, a revolutionary nanomaterial with excellent mechanical characteristics and a high specific surface area.^[^ ^165^ ^]^ For Gram‐negative *F. nucleatum*, the cell membrane is thinner due to the thinner peptidoglycan layer. Thus, GO is a kind of nanomaterial that acts as a nano‐knife that cuts the cell membrane directly and kills bacteria.^[^ ^166^ ^]^ Chen and colleagues employed a green strategy to prepare a polyetheretherketone (PEEK)‐polydopamine (PDA)‐GO (PAG) antibacterial coating that significantly decreased the amount of *F. nucleatum*.^[^ ^47^ ^]^ Furthermore, GO is known for its low cost, ease of synthesis, expedient modification, and good photothermal conversion performance.^[^ ^165^ ^]^ The surface plasmon band converts electromagnetic radiation to heat when GO is activated with a convenient near‐infrared (NIR) laser.^[^ ^167^ ^]^ The resulting heat can reach temperatures high enough to destroy bacterial cells. Wu and colleagues created a sandwich‐structured abutment out of GO wrapped in collagen.^[^ ^168^ ^]^ The optical density (OD) values of *F. nucleatum* decreased when GO was added and exposed to laser radiation. This result demonstrated that the photothermal effect imposed a significant effect on *F. nucleatum*.

GO's two‐dimensional sheet structure can exert its bactericidal effect by inducing cell damage or even cracking death through mechanical damage or chemisorption to bacterial cell membranes. Compared to antibiotics, GO exhibits long‐lasting bactericidal effects in suppressing bacterial growth on the surface of solid substrates (e.g., paper, water filtration membrane, skin, etc.) owing to their extraordinary stability.^[^ ^169^ ^]^

However, studies have demonstrated that exposure to GO in the gastrointestinal tract can lead to a decrease in species abundance and dysbiosis of the community structure in non‐pregnant mice and zebrafish.^[^ ^170^, ^171^ ^]^ The presence of GO also had an impact on intestinal probiotics. Exposure to GO resulted in the impairment of gastrointestinal tissues, which could potentially contribute to inflammatory consequences. In addition, it has been suggested that prolonged exposure to high concentrations of graphene may cause damage to the cell membrane and the destabilization of actin filaments and the cytoskeleton. Therefore, if GO systems are used for antimicrobial purposes in the colon, it is critical to protect against their potential toxicity.^[^ ^172^ ^]^ To improve biocompatibility, Zhou et al. prepared a carboxylated graphene‐β‐cyclodextrin/chlorhexidine acetate (GO‐COO‐β‐CD/CA), which possessed good biocompatibility as a result of the carrier's built‐in β‐CD molecules.^[^ ^173^ ^]^ Meanwhile, researchers developed a variety of methods for modifying GO with gelatin, polyethylene glycol (PEG), dextran (DEX), and fetal bovine serum (FBS) to improve biocompatibility.^[^ ^174^, ^175^, ^176^, ^177^ ^]^ In addition, when utilizing GO for photothermal action against bacteria at the CRC site, ensuring the specific targeting and elimination of *F. nucleatum* becomes a crucial challenge that needs to be addressed.

## 4 ORGANIC CHEMICALS

### 4.1 PSs

PS is a kind of photo‐excitable chemical that can be activated by low‐strength visible light to produce reactive oxygen species (ROS), such as superoxide anion (O^2−^), singlet oxygen (^1^O_2_), triplet oxygen (^3^O_2_), peroxide (O_2_^2−^), hydrogen peroxide (H_2_O_2_), hydroxyl radicals (·OH), and hydroxyl ions (OH^−^), to inactivate eukaryote or prokaryote cells for photodynamic therapy (PDT).^[^ ^178^, ^179^, ^180^, ^181^, ^182^ ^]^ PDT also outperforms conventional antibiotic treatments in terms of antibacterial efficacy against multidrug‐resistant bacteria.^[^ ^183^ ^]^ Several PSs, including chlorin e6 (Ce6), porphyrinic, toluidine blue O (TBO), riboflavin, curcumin, and others, have been explored.^[^ ^184^ ^]^ This section focuses on recent studies about the PDT‐based eradication of *F. nucleatum*.

Eick et al. designed an antimicrobial PDT (aPDT) system with riboflavin as the PS and a light‐emitting diode (LED) as the light source. When 0.25% or 3% hydrogen peroxide was used as a pretreatment, *F. nucleatum* counts were reduced by at least four orders of magnitude after 1 min of light exposure.^[^ ^185^ ^]^ Baek et al. developed a non‐invasive aPDT to investigate whether 650 nm LED could penetrate the soft tissue (3 mm) and activate TBO to eliminate *F. nucleatum*. The number of viable colonies decreased when 0.33 mm TBO and 60 mW cm^−2^ LED radiation were applied for 5 min via 3 mm‐thick artificial skin. Additionally, 3‐day‐old *F. nucleatum* biofilms were disrupted after 5 min of irradiation under the same experimental conditions.^[^ ^186^ ^]^

**FIGURE 4:**
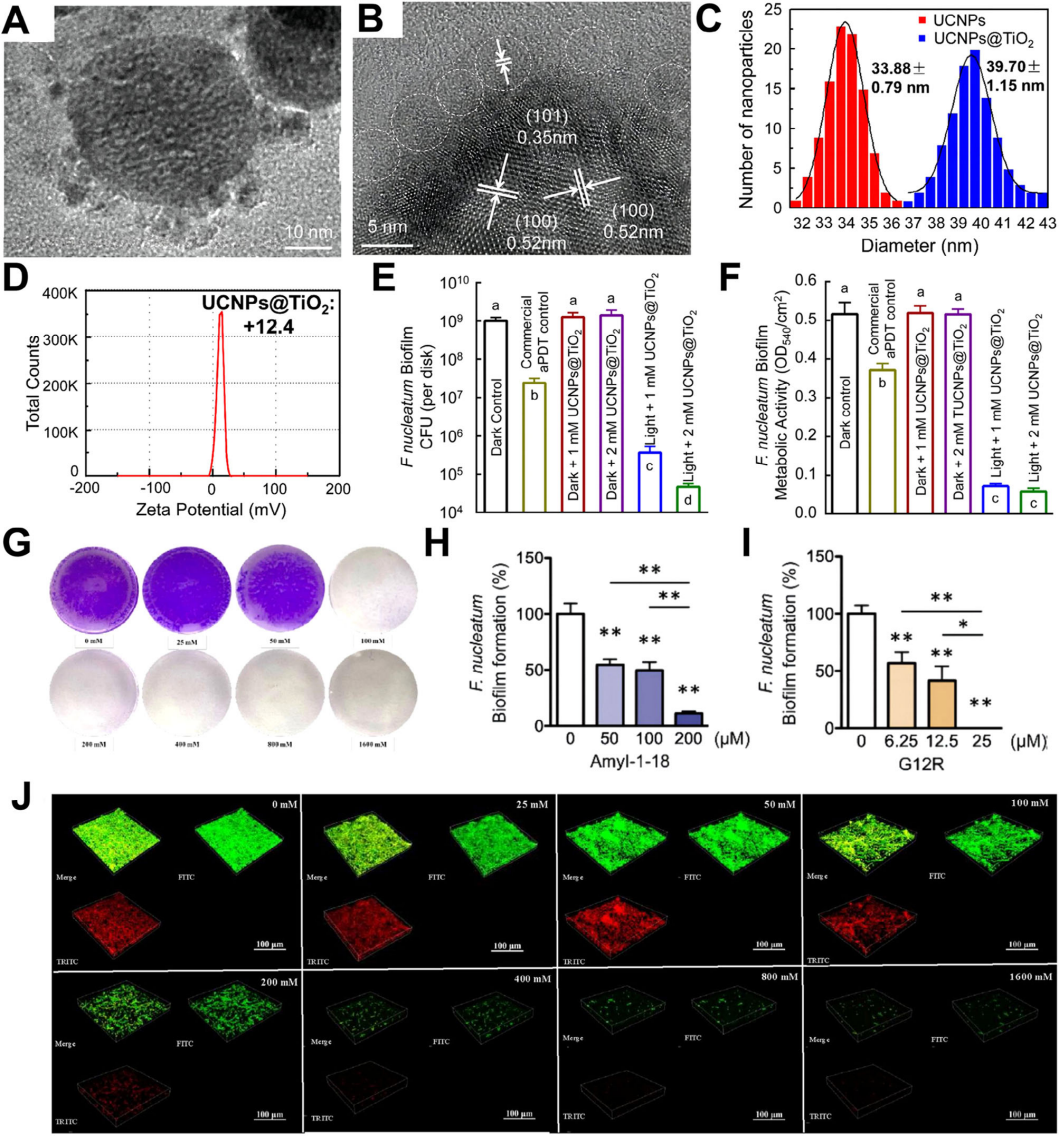
Physical properties and inhibition effect against *F. nucleatum* biofilms of UCNPs@TiO_2_: (A) TEM image of UCNPs@TiO_2_. (B) High‐resolution TEM image of UCNPs@TiO_2_. (C) Hydrodynamic diameters of UCNPs and UCNPs@TiO_2_. (D) Zeta potential of UCNPs@TiO_2_. (E) *F. nucleatum* biofilm CFU counts after four days. (F) Metabolism activity of four‐day *F. nucleatum* biofilms. Reproduced with permission.^[^ ^190^ ^]^ Copyright 2019, Elsevier. Inhibition of *F. nucleatum* biofilms by l‐lysine: (G) Effects of l‐lysine with different concentrations on the formation of *F. nucleatum* biofilms by CV staining. (J) CLSM‐3D live/dead fluorescence imaging of *F. nucleatum* biofilms. Reproduced with permission.^[^ ^201^ ^]^ Copyright 2022, Elsevier. Peptide effects on the development of a single‐species *F. nucleatum* biofilms: (H) AmyI‐1‐18 and (I) G12R. The remaining quantity of biofilm was measured using CV staining after incubation with each peptide. Reproduced with permission.^[^ ^205^ ^]^ Copyright 2020, Elsevier.

Excitation of small‐molecule PSs by visible light to produce ROS can kill *F. nucleatum* and remove the *F. nucleatum* biofilms. Visible light, however, cannot penetrate deeply into human tissue at the CRC site in the body.^[^ ^187^ ^]^ In comparison, NIR light is the most desirable trigger because it penetrates deeper into the tissue and causes minimal cellular damage with non‐invasive propagation.^[^ ^188^, ^189^ ^]^ Wang et al. synthesized a core‐shell structured β‐NaYF_4_: Yb^3+^, Tm^3+^@TiO_2_ (upconversion NPs (UCNPs)@TiO_2_) to inhibit *F. nucleatum* biofilms.^[^ ^190^ ^]^ UCNPs@TiO_2_ was observed to have a regular hexagonal shape by TEM (Figure 4A,B). The average diameter of positively charged UCNPs@TiO_2_ increased from 33.88 to 39.70 nm after being coated with TiO_2_ (Figure 4C,D). The CFU counts and 3‐(4,5‐dimethylthiazol‐2‐yl)‐2,5‐diphenyltetrazolium bromide (MTT) metabolic activity of 4‐day biofilms are shown in Figure 4E,F. When exposed to NIR irradiation, the CFU of all three species decreased when UCNPs@TiO_2_ was compared to all dark groups.

aPDT is a non‐invasive therapy modality that utilizes a light source to activate PSs enriched in the lesion, generating ROS that ultimately eradicates the target bacteria.^[^ ^191^ ^]^ Compared to conventional antibiotics, the ROS produced by PSs does not induce drug resistance because of the short light exposure, rapid membrane damage, and multiple locus disruptions without involving adaptive survival mechanisms. However, the produced ROS can affect normal cells and probiotics in the intestine site without selective inhibition against the pathogenic *F. nucleatum*. To address the selectivity of PSs for *F. nucleatum*, targeted PDT must be developed.^[^ ^192^ ^]^ This involves transporting PSs to the target cells using specific ligands that bind to appropriate receptors expressed at the target site. Furthermore, targeted receptors need to be expressed homogeneously on all targeted cells.^[^ ^193^, ^194^ ^]^ PSs complexed with peptides that have a high affinity for cell receptors can enhance bacterial accumulation via receptor‐mediated endocytosis. This approach could improve the recognition of PSs towards pathogenic flora in the gut and increase the specificity and efficiency of aPDT. However, finding specific targeting receptors on the surface of *F. nucleatum* becomes a major challenge. More PDT model designs and in vivo studies are thus required to address *F. nucleatum* proliferation and *F. nucleatum* biofilms at CRC sites.

### 4.2 AMPs

Over the last 20 years, more than 2,000 naturally occurring and synthesized AMPs have been produced as appealing alternatives to antibiotics.^[^ ^195^ ^]^ Cationic AMPs exhibit high affinity with microbial pathogens due to specific anionic components in the bacterial membrane, such as the LPS of Gram‐negative bacteria like *F. nucleatum*.^[^ ^196^ ^]^ The action mechanisms of AMPs include pore formation, the carpet model, and barrel‐stave model.^[^ ^197^, ^198^, ^199^, ^200^ ^]^ During these processes, AMPs accumulate in the bacterial membrane and create membrane pores to weaken the membrane, resulting in the collapse of the cell membrane structure, ultimately leading to leakage of the internal components of the bacteria. In this section, representative AMPs, including _L_‐Lysine, Nal‐P‐113, AmyI‐1‐18 rice peptide, LL‐37, and azurin, are introduced for application in *F. nucleatum* inhibition.

In the work by Sun et al., the gene expression profiles of *F. nucleatum* in both planktonic and biofilm states were identified. According to the transcriptome analysis and antibacterial activity tests, l‐Lysine was demonstrated to possess antibacterial and antibiofilm efficacy against *F. nucleatum* for the first time.^[^ ^201^ ^]^ The transcriptome analysis revealed that the genes involved in l‐Lysine metabolism distinguish between planktonic and biofilm stages. The MIC and MBC of l‐Lysine against *F. nucleatum* were 100 and 200 mm, respectively. Crystal violet (CV) staining and CLSM observation revealed that *F. nucleatum* biofilms can be efficiently inhibited by high doses of l‐lysine (Figure 4G,J). l‐Lysine may impede the growth of *F. nucleatum* due to its alkaline amino acid composition, which can make contact with bacterial membrane surfaces more accessible, and the NH^4+^ produced when it breaks down can alter the pH of the micro‐environment. Nal‐P‐113 (Ac‐AKR‐Nal‐Nal‐GYKRKF‐Nal‐NH_2_) is an AMP with negligible toxicity to humans within a specific concentration range that specifically replaces the histidine residues of P‐113 (AKRHHGYKRKFH‐NH_2_) with the bulky amino acid β‐naphthylalanine.^[^ ^202^ ^]^ Lin's study looked into the antibacterial and antibiofilm activity of Nal‐P‐113 against *F. nucleatum* in clinical studies.^[^ ^203^ ^]^ The bacterial growth curve implied that Nal‐P‐113 delayed the entry of *F. nucleatum* into the exponential growth phase, resulting in fewer bacteria. Furthermore, at a concentration of 20 µg mL^−1^, Nal‐P‐113 inhibited the formation of *F. nucleatum* biofilms. Wu et al. synthesized Nal‐P‐113‐PEG‐CSNPs by incorporating the AMP Nal‐P‐113 into PEGylated chitosan.^[^ ^204^ ^]^ When Nal‐P‐113 was added, the antibacterial activity of NPs improved significantly. Tabeta et al. also found that AmyI‐1‐18 rice peptide or its arginine‐substituted analog, G12R, could inhibit *F. nucleatum* in anaerobic conditions.^[^ ^205^ ^]^ They examined the effects of AmyI‐1‐18 and G12R peptides on the formation of single‐species biofilms. These peptides inhibited the formation of *F. nucleatum* biofilms in a dose‐dependent manner (Figure 4H,I). The MIC of G12R on *F. nucleatum* biofilm formation was 25 µm, which was lower than that of AmyI‐1‐18 (200 µm), indicating that G12R was more effective at inhibiting the formation of *F. nucleatum* biofilms than AmyI‐1‐18. The higher net charge of amino acids in G12R (+3) compared to AmyI‐1‐18 (+2) was hypothesized to increase the electrostatic interaction between the bacterial membrane and the G12R peptide, thereby increasing the antibacterial activity of AMPs. Furthermore, G12R was thought to possess more α‐helix than AmyI‐1‐18, further contributing to its antimicrobial activity.

Aside from being used alone, AMPs can also be employed in conjunction with antibiotics to disrupt the *F. nucleatum* biofilms. AMPs LL‐37 and Lactoferricin have been shown to enhance the anti‐biofilm actions of amoxicillin and clindamycin against facultative anaerobic biofilms. Metronidazole alone was ineffective at reducing facultative anaerobic biofilms, but when combined with LL‐37 and Lactoferricin, biofilm formation was significantly inhibited.^[^ ^206^ ^]^ In addition, AMPs are considered to be effective and promising candidates for cancer treatment. Naguib et al. investigated whether azurin, a promising anticancer drug derived from *Pseudomonas aeruginosa* (*P. aeruginosa*), could be immobilized on nano‐chitosan to boost its anticancer and antibacterial activities against gastrointestinal cancer and the bacteria that cause it.^[^ ^51^ ^]^ The MIC values for free azurin and nano‐azurin against *F. nucleatum* were determined to be 9.3 and 5.0 µg mL^−1^, respectively. The antibacterial process of azurin is mediated by direct contact with proteins or glycosylated proteins in the bacterial cell, resulting in protein activity disruption and eventual loss of bacterial membrane function.

Due to the emergence of antibiotic‐resistant bacteria, researchers are exploring the clinical potential of AMPs as promising alternatives to antibiotics. In contrast to conventional antibiotics, AMPs exhibit a wide range of antimicrobial effects with low cytotoxicity.^[^ ^207^ ^]^ This can be primarily attributed to their predominant mode of action, which involves targeting the bacterial cell membrane and exhibiting a limited propensity for the development of drug resistance. Among the metabolites released by the intestinal host, AMPs have the potential to modulate and maintain the stability of the intestinal microbiota.^[^ ^208^ ^]^ However, there are still certain limitations that persist in the progress and utilization of AMPs. These include challenges related to the extraction of such peptides from organisms and the significant expenses involved in their manufacturing process. More importantly, the bactericidal efficacy of AMPs varies depending on oxygen levels, reducing agents, and pH levels.^[^ ^209^ ^]^

As aforementioned, AMPs and amino acids are effective in inhibiting *F. nucleatum*. To successfully target *F. nucleatum* in the intestinal environment, however, AMPs must be modified to enhance selectivity and biocompatibility.^[^ ^210^ ^]^ To this end, Xiong et al. developed (PGA)_m_‐*r*‐(PHLG‐MHH)_n_, a pH‐sensitive, helix‐coil conformation transitional AMPs with randomly distributed negatively charged polyglutamic acid (PGA) and positively charged poly(γ‐6‐N‐(methyldihexylammonium)hexyl‐_L_‐glutamate) (PHLG‐MHH) residues.^[^ ^211^ ^]^ Under acidic pH, the positively charged PHLG‐MHH residues endow the AMP with specific targeting to *Helicobacter pylori* (*H. pylori*) while imposing no damage toward commensal bacteria or normal tissues. Similarly, modifying the appropriate residues on the AMPs may be a viable option for selectively targeting *F. nucleatum* in the gut. Research on the interplay of AMPs, intestinal probiotics, and organismal health, especially from an in vivo viewpoint, is now in its early stages. Further investigation is required to have a comprehensive understanding of the underlying mechanism through which intestinal probiotics interact with exogenous AMPs (Table 4).

**TABLE 4:** AMPs against *F. nucleatum* and *F. nucleatum* biofilms.

AMP	Antibacterial mechanism	Refs.
l‐Lysine	The alkaline amino acid composition of _L_‐Lysine makes contact with bacterial membrane surfaces more accessible, and the NH^4+^ produced when it breaks down can alter the pH of the micro‐environment.^[^ ^201^ ^]^	[201]
Nal‐P‐113	β‐naphthylalanine residues of Nal‐P‐113 could position themselves deeper into the bacterial and fungal cell membranes, making the AMP more efficient in disrupting the membranes.^[^ ^204^ ^]^	[203, 204]
G12R	G12R possesses a higher net charge of amino acids and more α‐helix, contributing to its antimicrobial activity.^[^ ^205^ ^]^	[205]
LL‐37 and Lactoferricin	AMPs LL‐37&Lactoferricin enhance the dispersion of matured biofilms, enhancing the antibacterial effect of amoxicillin.^[^ ^206^ ^]^	[206]
Azurin	Azurin is mediated by direct contact with proteins or glycosylated proteins in the bacterial cell, resulting in protein activity disruption and eventual loss of bacterial membrane function.^[^ ^51^ ^]^	[51]

### 4.3 Other compounds

Long carbon chain compounds are among the other compounds found in organic compounds. Organic materials with long carbon chains are often used in oral care products to inhibit the growth of *F. nucleatum*. For instance, in oral brush solutions, the addition of 0.05% cetylpyridinium chloride (CPC) to oral brush solutions was found to interfere with the development of *F. nucleatum* biofilms and affect their structure and viability. Furthermore, the tested solution exhibited bactericidal effects against *F. nucleatum* in both planktonic and mature biofilms.^[^ ^212^ ^]^ In Bryce's research, a single‐species biofilm of *F. nucleatum* was grown on nitrocellulose membranes for 72 h and exposed to solutions of Tween 80, cetyltrimethylammoniumbromide (CTAB), or sodium dodecyl sulfate (SDS) for 1, 5, or 10 min.^[^ ^213^ ^]^ Using a viability stain in conjunction with fluorescence microscopy, the number of viable and non‐viable bacteria “disrupted” from biofilms and those “remaining attached” was determined. CTAB and SDS were found to be more effective than Tween 80™ at disrupting *F. nucleatum* biofilms.

Long carbon chain materials, commonly used as surfactants, possess robust bactericidal properties in vitro or in the oral cavity. However, surfactants, as broad‐spectrum antimicrobial agents, can damage the intestinal flora and cause harm to the human body. To address this, targeting carriers can be introduced to refine the properties of long carbon chain compounds and enhance their biocompatibility, thus improving their ability to inhibit the proliferation of *F. nucleatum* in the vicinity of CRC. The context in this section introduces the components of mouthwash that primarily inhibit *F. nucleatum*. Because of this, there is still much research to be done before in vivo applications.

## 5 POLYMERS

Microorganisms typically possess cytomembranes that are negatively charged and composed of lipid layers and peptidoglycan. Consequently, polymers featuring a positively charged surface can augment their interaction with the bacterial surface. For in vitro assessments, polyhexanide (PHA) and polyhexamethylene biguanide (PHMB) are commonly employed for wound treatment as well as oral and ocular disinfection.^[^ ^52^, ^54^ ^]^ These agents exhibit promising outcomes in antimicrobial assays, effectively inhibiting *F. nucleatum* biofilms in vitro. More specifically, they have demonstrated a significant reduction in *F. nucleatum* survival relative to the control group, as confirmed by real‐time PCR.

**FIGURE 5:**
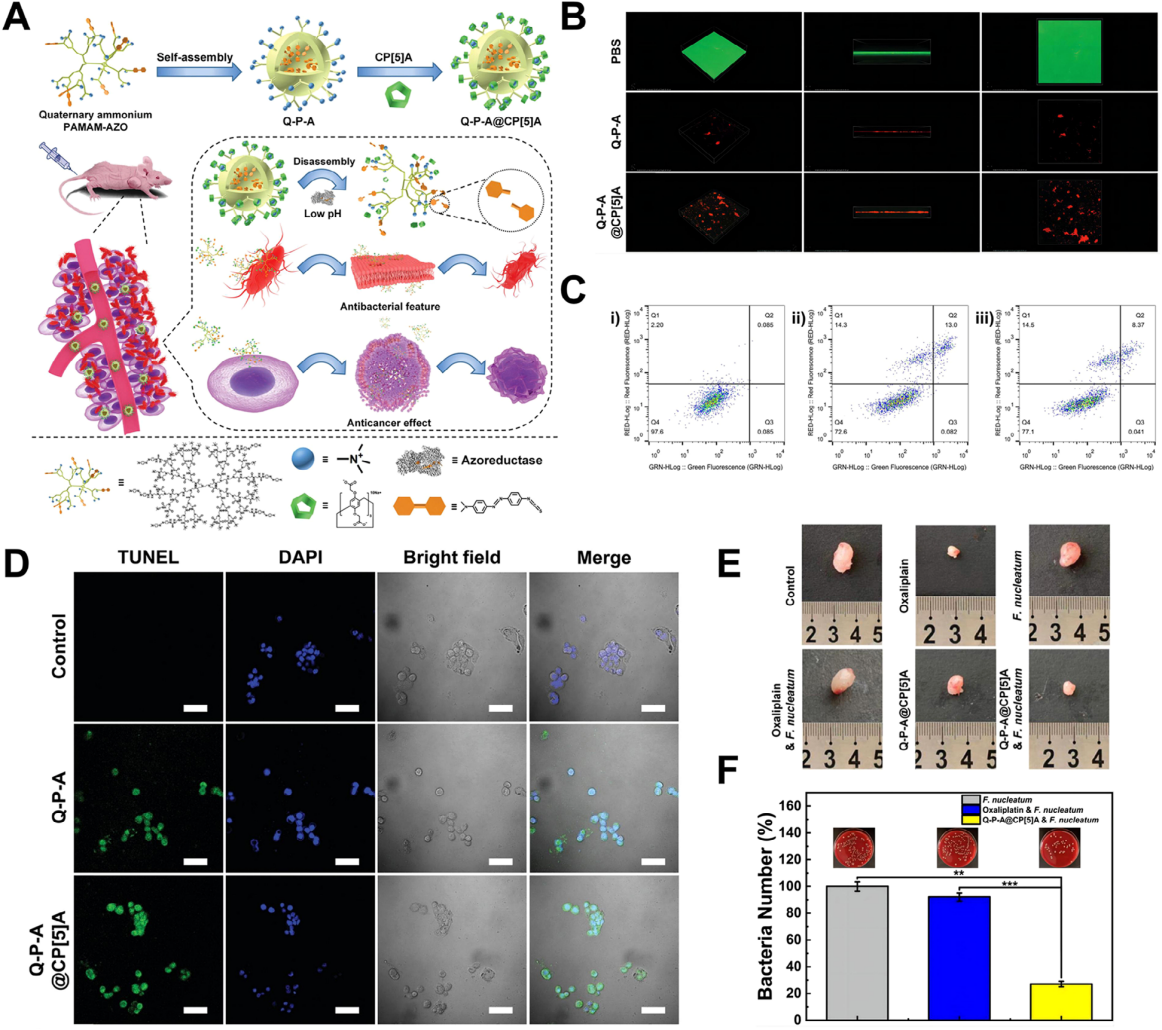
Design and therapeutic outcome of Q‐P‐A@CP[5]A for *F. nucleatum*‐induced drug‐resistant CRC: (A) Schematic illustration showing the construction of Q‐P‐A@CP[5]A and the treatment process for drug‐resistant CRC. (B) CLSM‐3D imaging of *F. nucleatum* biofilms following treatment with PBS, Q‐P‐A, and Q‐P‐A@CP[5]A (C) Flow cytometry analysis of *F. nucleatum*‐co‐cultured HT29 cells after being treated with PBS (i), Q‐P‐A (ii), and Q‐P‐A@CP[5]A (iii). (D) CLSM images of *F. nucleatum*‐co‐cultured HT29 cells after being treated with Q‐P‐A and Q‐P‐A@CP[5]A TUNEL‐positive cells exhibited green fluorescence, and DAPI‐stained nuclei showed blue fluorescence. Scale bar: 20 µm. (E) Representative photographs of HT29 tumor‐bearing nude mice treated by PBS (control), oxaliplatin, *F. nucleatum*, oxaliplatin, and *F. nucleatum*, Q‐P‐A@CP[5]A, and Q‐P‐A@CP[5]A and *F. nucleatum*. (F) Number of intratumor bacteria after various treatments. Inset: corresponding colony counts in tumor tissues. Reproduced with permission.^[^ ^53^ ^]^ Copyright 2021, Royal Society of Chemistry.

Polymers that self‐assemble into cationic NPs in an aqueous solution have been shown to exhibit enhanced bacterial membrane lysis and antibacterial capabilities due to an elevated positive surface charge in local regions.^[^ ^214^ ^]^ Our group created intelligent supramolecular cationic quaternary ammonium NPs called quaternary ammonium PAMAM‐AZO@CP[5]A (Q‐P‐A@CP[5]A), consisting of azobenzene (AZO)‐conjugated dendritic cationic quaternary ammonium PAMAM and host cyclic molecule carboxylatopillar[5]arene (CP[5]A), for drug‐resistant CRC caused by *F. nucleatum* (Figure 5A).^[^ ^53^ ^]^ In the CRC site, where reductase is abundant, the reduction of AZO led to the disassembly of Q‐P‐A@CP[5]A, which exposed the cationic quaternary ammonium group of PAMAM, effectively inhibiting the proliferation of *F. nucleatum*. As a result, the inhibition of *F. nucleatum* biofilms in vitro is shown in Figure 5B. The groups of Q‐P‐A and Q‐P‐A@CP[5]A disrupted the integrity of the biofilms significantly. In addition, Q‐P‐A and Q‐P‐A@CP[5]A can induce the apoptosis of HT29 (human colorectal carcinoma cells) with *F. nucleatum* infection, as demonstrated by Annexin V‐fluorescein isothiocyanate (Annexin V‐FITC)/propidium iodide (PI) double staining (Figure 5C) and terminal deoxynucleotidyl transferase dUTP nick end labeling (TUNEL) (Figure 5D). The results presented herein illustrated the potent efficacy of Q‐P‐A@CP[5]A inducing apoptosis of HT29 with *F. nucleatum* infection. In vivo experiments on mice infected with *F. nucleatum* revealed the development of chemoresistance to oxaliplatin (Figure 5E). However, Q‐P‐A and Q‐P‐A@CP[5]A groups exhibited significant inhibition of tumor growth, which can be attributed to the ability of Q‐P‐A@CP[5]A to impede *F. nucleatum* growth and thereby alleviate chemoresistance. Furthermore, as shown in Figure 5F, Q‐P‐A@CP[5]A exhibited commendable antibacterial activity, eradicating about 70% of *F. nucleatum* in CRC tumors.

The majority of anti‐*F. nucleatum* polymers rely on the abundance of positive charges to disrupt negatively charged bacterial cell membranes, thus achieving a broad‐spectrum antibacterial effect based on electrostatic interactions. This approach targets the fundamental chemical structure of bacteria, imposing little possibility for *F. nucleatum* to generate antibiotic resistance compared to antibiotics. In addition, polymer‐based antimicrobial materials possess significant abilities in combating antibiotic‐resistant bacteria and impeding the formation of bacterial biofilms.

However, cations would have a destructive effect on both the microbiota in the gut and normal biological tissues. It would be advantageous to introduce certain molecules (such as CP[5]A^[^ ^215^ ^]^, HA^[^ ^216^ ^]^) to enhance the biocompatibility of polymers. Additionally, nonbiodegradable polymers have the potential to remain and accumulate in the body, causing unwanted consequences such as inflammation and carcinogenesis.^[^ ^217^ ^]^ Therefore, utilizing a spontaneously biodegradable polymer is crucial for in vivo antibacterial purposes. Our previous research has focused on designing biodegradable antibacterial polymers based on cationic polyaspartamide derivatives.^[^ ^218^ ^]^ In addition to effectively eliminating bacteria, these biodegradable polymers possess adjustable antibacterial potency and can alleviate drug accumulation through their cleavable backbone.

## 6 INORGANIC–ORGANIC HYBRID MATERIALS

**FIGURE 6:**
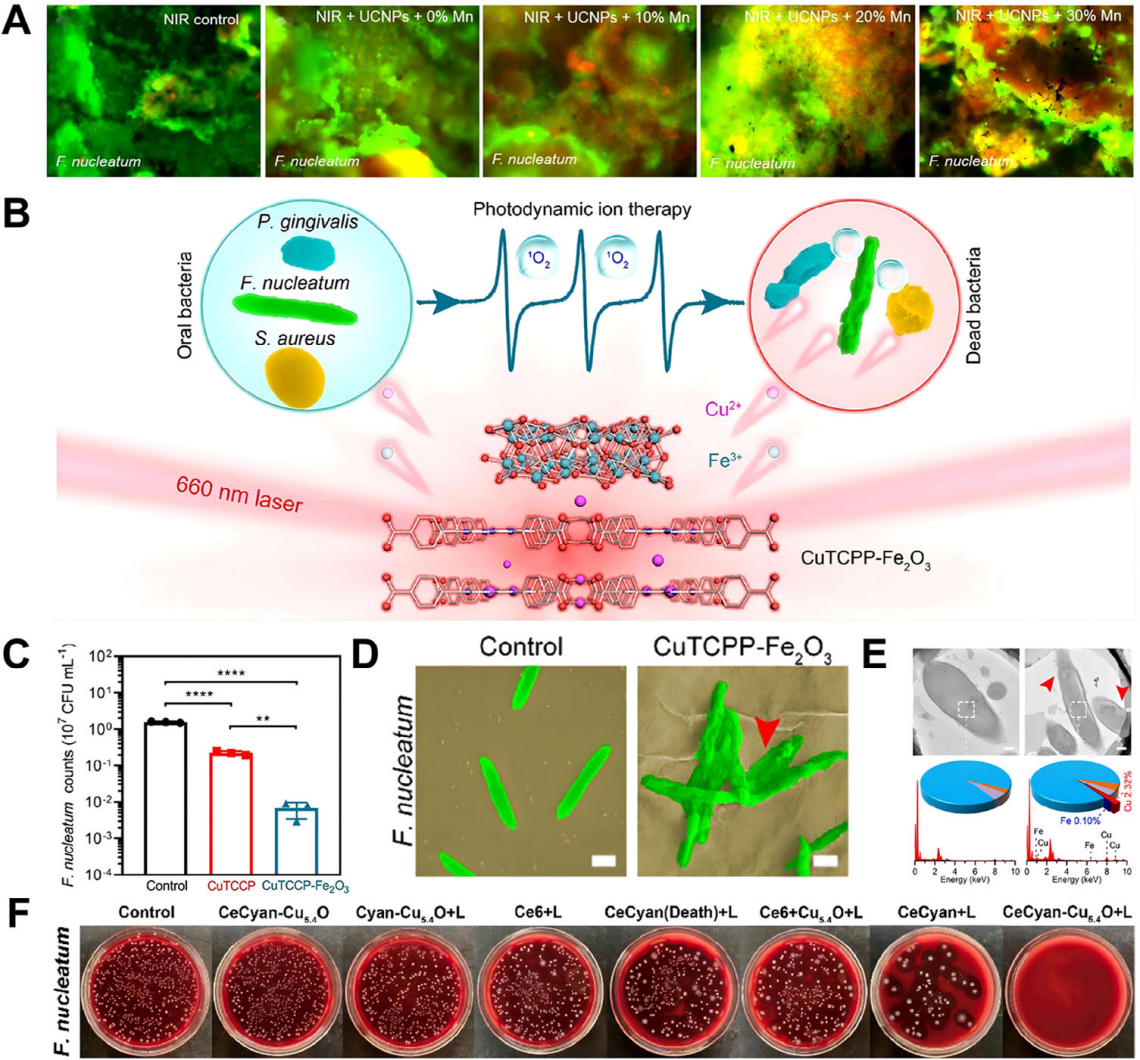
Effect of different proportions of Mn‐doped NaYF4@Ce6@silane on *F. nucleatum* biofilms under NIR irradiation: (A) CLSM‐3D images of live/dead cells of 4‐day biofilms of *F. nucleatum* NIR control, NIR + NaYF_4_@Ce6@silane, NIR + NaYF_4_‐Mn10%@Ce6@silane, NIR + NaYF_4_‐Mn20%@Ce6@silane, NIR + NaYF_4_‐Mn30%@Ce6@silane. Reproduced with permission.^[^ ^220^ ^]^ Copyright 2019, MDPI. Antimicrobial mechanism and efficacy of CuTCPP‐Fe_2_O_3_ against *F. nucleatum*. (B) Schematic diagram showing the antibacterial process of photodynamic ion therapy, which relies on the interaction of released ions and ROS. (C) CFU counts of *F. nucleatum* treated with CuTCPP and CuTCPP‐Fe_2_O_3_ for 20 min followed by 2‐h irradiation by 660 nm laser in the dark. (D) Scanning electron microscope images of *F. nucleatum* treated with CuTCPP‐Fe_2_O_3_ and 660 nm laser irradiation (scale bar: 500 nm). (E) TEM images and accompanying EDS curves of *F. nucleatum* treated with CuTCPP‐Fe_2_O_3_ and 660 laser irradiation. Scale bar: 200 nm. Reproduced with permission.^[^ ^57^ ^]^ Copyright 2021, American Chemical Society. The combined effect of CeCyan and Cu_5.4_O system on the PDT against *F. nucleatum*. (F) Representative photographs of *F. nucleatum* colonies following treatment in various ways (+ L indicates the process of exposure to a 660 nm laser at 200 mW cm^−2^). Reproduced with permission.^[^ ^58^ ^]^ Copyright 2022, Elsevier.

This section will introduce various innovative synergistic therapeutic modalities to combat the proliferation of *F. nucleatum*. Zhou and colleagues developed multi‐functional NPs called Fe_3_O_4_‐silane@Ce6/C6 MNPs, which co‐load PSs Ce6 and coumarin 6 (C6) into the Fe_3_O_4_‐silane core–shell structure.^[^ ^219^ ^]^ ROS produced by light exciting PSs effectively killed *F. nucleatum*, but Fe_3_O_4_‐silane@Ce6/C6 MNPs and silane@Ce6/C6 MNPs exhibited similar CFUs under light irradiation, suggesting that the incorporation of Fe_3_O_4_ into the MNPs did not affect the effect of aPDT on *F. nucleatum* biofilms. Besides, Zhou et al. prepared a composite nanomaterial UCNPs/Ce6 with red upconversion luminescence, and it presented remarkable aPDT performance against bacteria.^[^ ^220^ ^]^ Upon irradiation by a 980 nm laser, the ability of Mn‐doped NaYF_4_@Ce6@silane NPs to inhibit *F. nucleatum* growth improved as the Mn ion content in the system increased. Mn ions were doped to boost red light because the excitation location of Ce6 is in the red region, which resulted in an increase in the number of killed bacteria by PDT. (Figure 6A). Additionally, Wu and colleagues developed a photoresponsive ointment using an atomic‐layer Fe_2_O_3_‐modified 2D porphyrinic metal‐organic frameworks (MOF) (CuTCPP) system (CuTCPP‐Fe_2_O_3_) incorporated into a PEG matrix (Figure 6B). This biodegradable and biocompatible 2D MOF‐based heterostructure displayed antibacterial efficacy against *F. nucleatum*, *Porphyromonas gingivalis*, and *Staphylococcus aureus* by utilizing the synergistic effects of released ions and ROS.^[^ ^57^ ^]^ In particular, CuTCPP exhibited a moderate antibacterial rate of 85.51 ± 1.78% against *F. nucleatum*, as demonstrated in Figure 6C. In contrast, CuTCPP‐Fe_2_O_3_ showed a more potent antibacterial efficacy against *F. nucleatum* (99.57 ± 0.21%). The bactericidal effectiveness of CuTCPP was augmented by the inclusion of Fe_2_O_3_, which can be attributed to the combined action of released ions and ROS. TEM and energy‐dispersive X‐ray spectroscopy (EDS) were used to determine whether the Cu^2+^ and Fe^3+^ ions released from CuTCPP‐Fe_2_O_3_ could penetrate the bacteria. As a result, CuTCPP‐Fe_2_O_3_‐treated *F. nucleatum* displayed a fractured morphology compared to the control group (Figure 6D), and specific Cu and Fe elements were observed on the cell wall (Figure 6E). Since the released Cu^2+^ and Fe^3+^ ions from CuTCPP‐Fe_2_O_3_ entered the bacteria and caused sufficient mortality, the results fully supported the hypothesis. Combining ROS and the release of metal ions in a system provided more insight into an antibacterial strategy and has great advantages for anti‐*F. nucleatum*.

In Wang's study, by combining CeO_2_ with Ce6, a hybrid nanomaterial with antibacterial and anti‐inflammatory capabilities was created, which outperformed the standard aPDT.^[^ ^221^ ^]^ The improved antibacterial efficacy of CeO_2_@Ce6 could be attributed to the production of ROS by aPDT as well as the inherent antibacterial activity of CeO_2_. However, the levels of ROS produced exceeded the bactericidal requirements, resulting in local ROS buildup that exacerbated inflammation. Fortunately, CeO_2_ exhibited both superoxide dismutase (SOD) and catalase (CAT) mimetic activities, enabling the CeO_2_‐containing nanomaterials to scavenge redundant ROS owing to the switch between Ce (III) to Ce (IV) valence states. Most importantly, the ROS reduction was detected within several minutes of aPDT, indicating that this delayed ROS reduction would not affect the anti‐*F. nucleatum* efficiency of aPDT in the initial stage.

Qu et al. proposed a synergistic functional system (CeCyan‐Cu_5.4_O) by loading Ce6 and ultrasmall Cu_5.4_O NPs (Cu_5.4_O USNPs) into spontaneous oxygen‐producing cyanobacteria, which was employed to enhance the efficacy of PDT under a hypoxic microenvironment against anaerobic *F. nucleatum* and alleviate inflammation following anaerobe infections.^[^ ^58^ ^]^ By modifying the ratio between the oxidative stress‐removal component (Cu_5.4_O USNPs) and the antibacterial component (Ce6), it can achieve rapid bacteria killing and biofilm elimination, as well as effective alleviation of oxidative stress. The O_2_ production in the CeCyan‐Cu_5.4_O + L group (+ L refers to the process of irradiation under a 660 nm laser at 200 mW cm^−2^) significantly improved the antibacterial property in such a hypoxic environment (Figure 6F). Meanwhile, after establishing the anaerobic infectious keratitis model, remarkable turbidity was observed in the cornea. The cornea in the CeCyan‐Cu_5.4_O + L group regained its transparency in three days, implying the fundamental remission of inflammation. The prompt inflammation elimination function of CeCyan‐Cu_5.4_O should be attributed to the ROS catalytic degradation ability of Cu_5.4_O after bacterial killing.

This section outlines the strategies involving the combination of metals with ROS, as well as the combination of ROS scavengers with ROS. First, in metal ions‐coordinated ROS work, ROS and metal ions exhibit dual antibacterial effects against *F. nucleatum*. Second, the combination of PS with ROS scavengers provides a better solution to the problem of ROS‐induced damage to normal cells and tissues. Third, in a hypoxic environment, PSs can transport the oxygen‐supplying module to the lesion site to produce more ROS to combat CRC‐associated *F. nucleatum*. While using cyanobacteria as oxygen supply modules is a promising strategy, it has yet to be explored in vivo. However, with hybrid materials, there is a possibility that one factor may dominate all actions, thereby weakening the other. When the hybrid materials reach the intestinal site, either one of the factors in the hybrid materials is weakened or fails, causing a burden on the human body. Therefore, future antibacterial systems with free switchable hybrid materials will be designed, allowing for the free choice of the antibacterial approach depending on the actual inhibition against *F. nucleatum* (Table 5).

**TABLE 5:** Inorganic–organic hybrid materials against *F. nucleatum* and *F. nucleatum* biofilms.

Inorganic–organic hybrid material	Main components	Antibacterial mechanism	Refs.
Fe_3_O_4_‐silane@Ce6/C6 MNPs	Ce6, C6, Fe_3_O_4_‐silane core–shell structure	ROS produced by light exciting Ce6, C6.^[^ ^219^ ^]^	[219]
UCNPs/Ce6	Mn, NaYF_4_:Yb^3+^,Er^3+^,Ce6, silane	Mn ions were doped to boost red light by exciting Ce6, which increased the number of killed bacteria by PDT.^[^ ^220^ ^]^	[220]
CuTCPP‐Fe_2_O_3_	Atomic‐layer Fe_2_O_3_,CuTCPP	Combining ROS and the release of Cu^2+^ and Fe^3+^ ions increases bactericidal effectiveness.^[^ ^57^ ^]^	[57]
CeO_2_@Ce6	CeO_2_,Ce6	ROS production by Ce6 and the inherent antibacterial activity of CeO_2_ collaborative improved antibacterial efficacy.^[^ ^221^ ^]^	[221]
CeCyan‐Cu_5.4_O	Ce6, Cu_5.4_O USNPs, cyanobacteria	Cyanobacteria provide oxygen in hypoxic locations; Cu_5.4_O USNPs and Ce6 achieve rapid bacteria killing, as well as effective alleviation of oxidative stress.^[^ ^58^ ^]^	[58]

## 7 BACTERIOPHAGES

*F. nucleatum*, a gut microbial species, has been linked to CRC progression and chemoresistance. While antibiotic treatment is one method of eliminating harmful bacteria, this relatively crude method may also remove beneficial bacterial species.^[^ ^222^ ^]^ Despite the outstanding feedback of the above‐mentioned approaches on the inhibition of *F. nucleatum* and *F. nucleatum* biofilms, the relative insufficiency of antibacterial specificity is the main obstacle to applications in the intestinal tract. Therefore, researchers are exploring strategies that selectively manipulate certain bacterial species within the gut microbiota, leading them to investigate bacteriophages.^[^ ^223^, ^224^ ^]^ Bacteriophages are viruses that have all the common viral properties and exhibit tight host‐cell specificity.^[^ ^225^ ^]^ Previous research mainly focused on the bacteriophages ɸFunu1 and ɸFunu2 associated with *F. nucleatum* subsp. animalis strain 7‐1. Meanwhile, bacteriophage signatures in sequenced *F. nucleatum* genomes were also forecasted and compared using computational approaches.^[^ ^226^ ^]^ Here, we present several bacteriophages that offer novel solutions for combating *F. nucleatum*.

**FIGURE 7:**
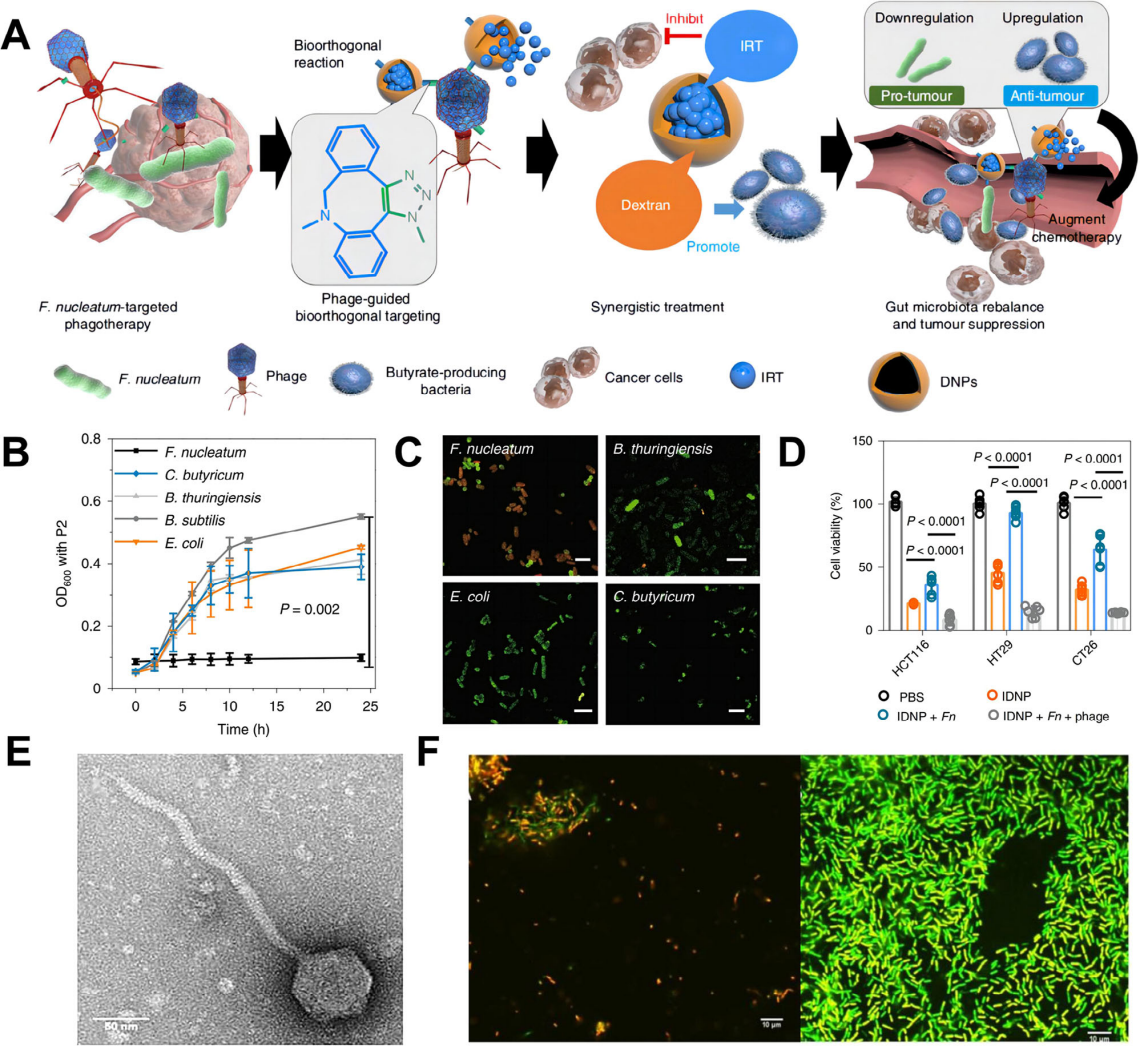
P2 phage inhibiting *F. nucleatum* and suppressing *F. nucleatum*‐induced drug‐resistant CRC in coordination with IDNP. (A) Schematic illustration showing the detailed process of the phage‐guided biotic‐abiotic hybrid nanosystem for tumor suppression. (B) In vitro lysis of various bacterial species by P2 phages. (C) CLSM images of various species of bacteria after being bound with P2 phages. Bacteria with green fluorescence were labeled with FITC, whereas phages with red fluorescence were stained with RhB. (D) In vitro anticancer impact of phage and chemotherapy (IDNP) against *F. nucleatum*‐co‐cultured CRC cells. Results for the mice bearing orthotopic CT26luc tumors. Reproduced with permission.^[^ ^227^ ^]^ Copyright 2019, Springer Nature. Morphology and anti‐biofilm capability of FNU1. (E) TEM image of FNU1 showing the morphology and size of Siphoviridae bacteriophage. (F) CLSM images of SYBR gold and PI staining following FNU1 bacteriophage treated (left) and untreated (right) *F. nucleatum* biofilms. Reproduced with permission.^[^ ^60^ ^]^ Copyright 2019, Springer Nature.

Zhang et al. identified a phage strain (P2) from human saliva that lyses *F. nucleatum* selectively. Irinotecan (IRT) was encapsulated within DEX NPs (DNPs) to create IRT‐loaded DNPs (IDNPs) and covalently linked azodibenzo‐cyclooctyne (DBCO)‐modified IDNPs (D‐IDNPs) to azide‐modified phages (A‐phages), thus constructing a phage‐guided biotic–abiotic hybrid nanosystem.^[^ ^227^ ^]^ A‐phage showed significant tumor accumulation, and intratumoral *F. nucleatum* was efficaciously scavenged by oral administration of the biotic‐abiotic hybrid nanosystem (Figure 7A). P2 inhibited the growth of *F. nucleatum* exclusively while sparing other bacteria from being harmed (Figure 7B). A fluorescence labeling method confirmed the same result. Rhodamine‐B (RhB)‐labeled P2 phages adhered well to FITC‐labeled *F. nucleatum* (Figure 7C). Chemotherapy resistance induced by *F. nucleatum* was effectively reversed in CRC cells (CT26 (mouse colon adenocarcinoma cells), HCT116, and HT29) following P2 phage treatment (Figure 7D). After implanting CT26luc cells into the caecum of BALB/c mice, the mice were subjected to various treatments. The mice given both A‐phages and D‐IDNPs had the smallest tumor volumes as monitored by the bioluminescence imaging. The results demonstrated that the phage‐guided biotic‐abiotic hybrid nanosystem could improve CRC chemotherapy by precisely regulating *F. nucleatum* in the intestine. Tucci et al. described the complete genomic and morphological characterization of a novel lytic bacteriophage FNU1 with a tail length of ≈310 nm and an icosahedral head diameter of ≈88 nm, which can disrupt existing *F. nucleatum* biofilms (Figure 7E).^[^ ^60^ ^]^ When biofilm viability was evaluated following treatment with bacteriophage FNU1, the bacteriophage‐treated biofilms displayed few cells, the majority of which were red/yellow, suggesting structurally damaged membranes, and only a very small number of cells with intact membranes (green) clumped together (Figure 7F).

Bacteriophages, which directly interact with bacteria and inject viral genes into them, have the ability to effectively replace bacterial genes and eliminate infections. Bacteriophages can prevent bacterial reproduction, producing more bacteriophages, and they specifically target certain bacterial strains without affecting other bacteria, including probiotics. This targeted approach reduces the risk of drug resistance development.^[^ ^228^, ^229^ ^]^ Therefore, it is expected that bacteriophage treatment would result in less or negligible adverse effects compared to traditional antibiotics, even when administered at doses exceeding the therapeutic level. However, one drawback of bacteriophages is their relatively weak antibacterial effect. The prevalence of this phenomenon can be mostly attributed to the ability of bacteria to employ the clustered regularly interspaced short palindromic repeats (CRISPR) system as a defense mechanism against bacteriophages.^[^ ^230^, ^231^ ^]^ Unfortunately, there remains a dearth of efficacious strategies to circumvent this resistance. The antibacterial activity of bacteriophages will need to be improved in the future to achieve optimal results on their own. Furthermore, because bacteriophages primarily consist of proteins and DNA or RNA, they are vulnerable to degradation when exposed to the human body, including the stomach and liver, as well as when encountering the animal immune system. These factors pose challenges in the clinical implementation of bacteriophages.

The improvement in effectiveness of bacteriophage therapy can be aimed at the synergistic employment of bacteriophages and antibiotics. In a preclinical study, the combination of bacteriophage ɸFG02 with ceftazidime exhibited more significant efficacy in combating *Acinetobacter baumannii* bacteraemia compared to a single treatment, implying the superior potential of the combined bacteriophage and antibiotic treatments.^[^ ^232^ ^]^

## 8 PROBIOTICS

Microorganisms that are classified as probiotics are those that coexist peacefully with their human hosts. Probiotics can regulate biological processes with beneficial impacts on health when applied in appropriate amounts.^[^ ^61^ ^]^ Probiotics have been found to have various biological benefits, such as antimicrobial activity. However, research in the field of anti‐*F. nucleatum* is still in its infancy and requires further discussion.^[^ ^233^ ^]^ This section attempts to generalize current knowledge on the ability of probiotics to inhibit *F. nucleatum*. The probiotics that have been shown to inhibit *F. nucleatum* include Kimchi 44 and Kimchi 71, *Lactobacillus salivarius* subsp. *salicinius* AP‐32 (*L. salivarius* subsp. *salicinius* AP‐32), *Bifidobacterium animalis* subsp. *lactis* CP‐9 (*B. animalis* subsp. *lactis* CP‐9), *Lactobacillus paracasei* ET‐66 (*L. paracasei* ET‐66), *L. gasseri HHuMIN* D, and *Lactobacillus reuteri* AN417 (*L. reuteri* AN417).

Yoon et al. explored 360 probiotic bacteria isolated from kimchi and 110 probiotic bacteria isolated from cheese.^[^ ^63^ ^]^ Among these, Kimchi 44 and 71 were found to have antibacterial effects and to be more effective against *F. nucleatum* than *Lactobacillus rhamnosus* GG (LGG). Ho and colleagues confirmed the effects of mixed pills with live probiotics containing strains *B. animalis* subsp. *lactis* CP‐9, *L. paracasei* ET‐66, and *L. salivarius* subsp. *salicinius* AP‐32 on the oral pathogen.^[^ ^234^ ^]^ The in vitro survival of pathogenic *F. nucleatum* declined dramatically after treatment with live probiotic pills. The survival rate of *F. nucleatum* in the group of live probiotics fell to 5.77%, which was significantly lower than the placebo group. Hwang et al. isolated *L. gasseri* HHuMIN D from the human body and tested its antibacterial capabilities.^[^ ^64^ ^]^ The culture supernatant of *L. gasseri* HHuMIN D inhibited *F. nucleatum* by 89% at a 5% dilution. H_2_O_2_, generated by *L. gasseri* HHuMIN D, is a weapon against *F. nucleatum*. Bae and colleagues investigated the antibacterial properties of *L. reuteri* AN417 culture supernatant (LRS) on *F. nucleatum* in their study.^[^ ^235^ ^]^ The concentration of LRS was varied at 10%, 20%, 30%, and 40% (v/v) to determine its effect on *F. nucleatum* growth. The growth of *F. nucleatum* was found to be dependent on the concentration of LRS, with 40% (v/v) LRS causing significant inhibition of growth. The inhibitory effect remained consistent for up to 48 h.

Numerous studies have examined the efficacy of probiotics against *F. nucleatum*; however, the precise molecular mechanisms underlying this inhibition remain unclear and hypothetical. Research suggests that certain probiotics can interact with intestinal epithelial cells, leading to a competitive inhibition of binding sites. As a result, this interaction hinders the adhesion and colonization of intestinal pathogens. For instance, *Lactobacillus helveticus* has been observed to possess the ability to non‐specifically bind to host cells.^[^ ^236^ ^]^ In addition, studies have demonstrated that the probiotic *Lactobacillus* cells can create a barrier effect against *Salmonella* by adhering to epithelial cells in different regions of the mouse gut.^[^ ^237^ ^]^ Similar results were observed in vitro, where probiotic bacteria adhered to the brush boundaries of enterocyte‐like Caco‐2 cells, thereby inhibiting the adhesion and entry of *Salmonella* into the cells.^[^ ^238^ ^]^ Through both in vivo and in vitro experiments, probiotics have demonstrated their effectiveness in controlling pathogens at the intestinal site. The use of probiotics as a non‐pharmaceutical approach to inhibit the growth of harmful bacteria by modulating the balance of the gut microbiota is well established. When consumed in suitable amounts, probiotics typically do not pose any significant risk to human health. However, the effectiveness of live bacterial preparations can be diminished by changes in the surrounding environment, which can result in an uncertain survival rate of probiotics and compromise the quality of the product. Additionally, most probiotics that enter the digestive tract are susceptible to the influence of gastric acid and bile salts. As a result, only a limited number of viable bacteria reach the intestinal tract and successfully establish colonization, which hinders their efficacy in the intestinal environment. Therefore, it is crucial to select appropriate encapsulation methods to ensure the successful delivery of probiotics to the colon. Jiang et al. employed a coaxial electrostatic spinning technique to encapsulate *Lactobacilli* probiotics within polylactic acid nanofibers. Additionally, oligofructose (FOS) was incorporated as a prebiotic to augment the survival of the probiotics in the gastrointestinal tract. The resulting nanofiber membrane exhibited desirable hydrophobicity and biocompatibility, while the encapsulated lactic acid bacteria showed excellent activity.^[^ ^239^ ^]^ In a study carried out by Hussein et al. focusing on anti‐CRC strategies, they utilized *Lactobacillus acidophilus* ghosts (LAGs) that were modified with prodigiosin to create a novel delivery system derived from bacteria (referred to as prodigiosin‐LAGs). This approach aimed to harness the combined capabilities of LAGs and prodigiosin. The growth of HCT116 cells was effectively suppressed by prodigiosin‐LAGs, as observed at the cellular level. In terms of molecular characteristics, the expression levels of apoptotic caspase 3 and P53 biomarkers in HCT116 intracellular proteins were notably increased, while the expression of the anti‐apoptotic B‐cell lymphoma 2 (Bcl‐2) decreased upon treatment with prodigiosin‐LAGs.^[^ ^240^ ^]^

Currently, the anti‐*F. nucleatum* probiotic systems are predominantly limited to in vitro and oral models. However, further in vivo research is necessary to demonstrate the efficacy of probiotics in inhibiting *F. nucleatum* specifically at the CRC site.

## 9 VACCINES

Vaccination has made enormous strides over the last 40 years. The response to the COVID‐19 epidemic has accelerated this progress even further. Vaccines play a significant influence in combating bacterial infections by triggering the immunological system of the human body to identify and eliminate harmful agents.^[^ ^65^ ^]^ This immune response also recognizes and neutralizes any future encounters with the same microorganisms. Therefore, vaccines can be used to stimulate the immune system to target harmful bacteria like *F. nucleatum* while leaving beneficial gut microbes alone. In this regard, several promising *F. nucleatum*‐targeted vaccines are being developed, which will aid in expanding the anti‐*F. nucleatum* pathway.

Huang et al. discovered that inactivated *F. nucleatum*‐based vaccines can elicit a systemic immune response. Mice immunized with inactivated *F. nucleatum* demonstrated a significant reduction in the progression of abscesses.^[^ ^241^ ^]^ Furthermore, the researchers proposed an immunization approach that targets the FomA outer membrane protein of *F. nucleatum*. The inhibition of bacterial co‐aggregation and biofilm formation mediated by *F. nucleatum* was significantly reduced when FomA was neutralized.^[^ ^34^ ^]^ Zhang et al. investigated the effectiveness of *F. nucleatum*‐antioxidant protein alkyl hydroperoxide reductase subunit C (*F. nucleatum*‐AhpC) as a potential vaccine for CRC.^[^ ^242^, ^243^ ^]^ They used western blot analysis to demonstrate that antibodies found in the serum of CRC patients could recognize the *F. nucleatum*‐AhpC recombinant protein specifically. The researchers discovered that mice who received an intraperitoneal (*i.p*.) injection of AhpC/alum exhibited immunity against *F. nucleatum* infection and showed greater protection compared to those who were immunized with AhpC alone. Specifically, mice immunized with AhpC/alum were protected from *F. nucleatum* infection in 74.8% of cases, whereas mice immunized with AhpC alone were protected in only 53.6% of cases. Flow cytometric analysis was used to further identify the viability of *F. nucleatum*. The survival rate of *F. nucleatum* considerably decreased upon exposure to a low anti‐AhpC titer (at a 1:4 dilution) (77% vs 32.5%), and only a negligible amount of bacteria survived (only 2.1%) when exposed to a high titer (at a 1:1 dilution). These results suggested that rather than having a bactericidal impact, antibodies to AhpC might impede the growth of *F. nucleatum* by functioning as an antibody‐based AhpC inhibitor.

Vaccines have shown promise as a strategy for combating bacteria that are resistant to antibiotics and offer a new solution for tackling pathogenic *F. nucleatum*. Live‐attenuated vaccines have been available since the last century and are created by weakening viruses or bacteria in laboratory conditions. This process simulates natural infections, resulting in either no or very mild disease while still inducing immunity.^[^ ^243^ ^]^ Furthermore, the remarkable specificity of vaccinations provides protection to the host against specific infections while leaving non‐pathogenic microorganisms, such as gut probiotics, unharmed.

Despite the proposed vaccine formulations for *F. nucleatum* prevention, successful phase III clinical trials have yet to be conducted. Moreover, even though vaccination can trigger specific immune reactions, such as human versus T‐cell responses, certain strains of *F. nucleatum* are still able to evade immune killing during their intracellular phase. Thus, further research is needed to refine the anti‐*F. nucleatum* vaccine and better facilitate host‐pathogen interactions. To improve the antimicrobial properties of the *F. nucleatum* vaccine, the incorporation of an immunological adjuvant can effectively boost the immune response to the antigen.^[^ ^244^ ^]^ Adjuvants have the potential to extend the presence of antigens, amplify co‐stimulatory signaling, and induce non‐specific lymphocyte proliferation, thereby enhancing the immunotherapeutic efficacy of the vaccine.

## 10 CONCLUSIONS AND OUTLOOK

CRC is the third most prevalent type of cancer worldwide and the leading cause of cancer‐associated mortality. Research has shown that the presence of *F. nucleatum* in tumor tissues plays a significant role in the initiation, progression, and spread of CRC. Additionally, studies have pointed toward its connection with chemotherapy resistance, a weakened immune response, and an unfavorable prognosis.^[^ ^245^ ^]^ Numerous studies have investigated the potential of *F. nucleatum* as a target for CRC treatment. However, developing an efficient antibacterial system to specifically eliminate *F. nucleatum* in the CRC sites remains a great challenge. This review focused on and summarized the currently employed materials for targeting and eliminating pathogenic *F. nucleatum*, including natural extracts, inorganic chemicals, organic chemicals, polymers, inorganic–organic hybrid materials, bacteriophages, probiotics, and vaccines. We especially highlighted the unique advantages and potential pitfalls of each material and possible future improvements for the more effective elimination of *F. nucleatum*. We also briefly discussed the critical elements that should be paid attention to while an antibacterial system is applied to the control of intestinal flora. In experiments aimed at inhibiting *F. nucleatum* proliferation and disrupting *F. nucleatum* biofilms, various materials have achieved remarkable outcomes in vitro and in vivo. They have demonstrated the excellent performance of novel antimicrobial agents against *F. nucleatum* and suggested new pathways to substitute traditional antibiotics in the post‐antibiotic era. Nevertheless, with the increasing demand for drug resistance, biocompatibility, and specificity of antimicrobial materials for practical applications, the application of anti‐*F. nucleatum* tools will face both opportunities and challenges.

- First, drug resistance occurs when the same dose of a drug is less effective over time due to prolonged use against the same pathogens. Misuse of antibiotics in the past has led to drug resistance in bacteria, making them ultimately untreatable. In experiments aimed at inhibiting drug resistance to *F. nucleatum*, certain strategies have utilized new natural extract molecules, metal ions (e.g., silver ions), ROS, etc. These approaches have shown promise in preventing or overcoming drug resistance in *F. nucleatum*, and further research in this area is necessary to develop effective treatments against this pathogen.
- There are certain limitations associated with the current strategies employed against *F. nucleatum*. The in vivo bioavailability of natural products is still unknown, despite their demonstrated inhibition of *F. nucleatum* in vitro. The mechanism of bacterial death induced by PSs involves the oxidation of bacterial surface proteins, leading to membrane rupture. Nevertheless, this approach lacks selectivity as it may indiscriminately damage normal cells along with the targeted bacteria. Metal‐based materials possess the ability to adhere to the cell membrane through Coulomb force, facilitating their penetration into the cell and ultimately leading to bacterial death. Inevitably, metal ions can also penetrate normal cells, resulting in elevated intracellular ROS levels that can be detrimental to human health. AMPs exhibit distinct characteristics compared to conventional antibiotics since they effectively eliminate bacteria by perforation while also demonstrating a reduced propensity for inducing drug resistance. However, peptide drugs have disadvantages such as a short half‐life and susceptibility to hydrolysis, which necessitates their rational design. Bacteriophages trigger bacterial lysis by specifically inhibiting the synthesis of bacterial cell wall while undergoing extensive self‐replication. Nevertheless, once the bacteriophage gains entry into the host organism, it triggers the production of targeted neutralizing antibodies by the host immune system, thereby impeding its capacity to infect bacterial cells. Taken as a whole, it becomes apparent that current anti‐*F. nucleatum* systems continue to possess numerous limitations, particularly in the treatment of *F. nucleatum*‐resident CRC, which should be considered comprehensively in the future.
- Antimicrobial materials that specifically target the pathogenic *F. nucleatum* in the CRC sites have attracted a lot of scientific attention. As expounded upon in this review, various antimicrobial systems have demonstrated exceptional efficacy in inhibiting *F. nucleatum* in in vitro experiments, oral models, and murine models. Ensuring that antimicrobial materials reach the CRC sites without adversely affecting the intestinal microbiota is a crucial consideration that warrants further investigation. Nonetheless, it is vital to recognize that every treatment alternative possesses its own set of possible benefits and drawbacks. To mitigate the risk of immune rejection and minimize harm to healthy tissues and microbiota, choosing an endogenous antibacterial approach to control *F. nucleatum* is a prudent decision. Fortunately, the emergence of probiotic therapy may serve as a viable solution to these obstacles in the future. Probiotics have several mechanisms for their antibacterial action; this includes the creation of inhibitory substances, such as bacteriocins and hydrogen peroxide, obstructing adhesion sites, and competing for nutrients.^[^ ^246^, ^247^, ^248^, ^249^, ^250^ ^]^ Studies have been done to determine how probiotics interact with bacteria. The precise molecular pathways of probiotics for inhibiting pathogenic strains, however, remain largely unknown and subject to speculation. Despite this, probiotics are considered safe for the human body and are known to protect the intestinal tract.^[^ ^61^ ^]^ Additional research is necessary to enhance the effectiveness of probiotics by improving their composition and determining optimal routes of administration.
- *F. nucleatum* is a bacterium that can specifically colonize CRC tissues, forming biofilms and even invading tumor cells. This makes them resistant to antibiotics and difficult to remove, leading to the promotion of carcinogenesis. *F. nucleatum* adheres to intestinal epithelial cells via specifically secreted FadA and Fap2, completing colonization and promoting tumorigenesis. Therefore, directed attenuation of FadA or Fap2 could prevent transmembrane signals and inhibit tumorigenesis induced by *F. nucleatum*. In addition to targeted attenuation, bacteriophages have been used as bio‐therapeutics to achieve specific clearance of *F. nucleatum* in vivo. Bacteriophages are viruses that infect and kill bacteria, making them a potential strategy for targeted control of *F. nucleatum*. Therefore, the development and exploration of bacteriophage therapy should be further pursued as a strategy for targeted control of *F. nucleatum*. This could lead to the development of more effective and specific treatments for CRC.
- Generally, there are several issues among these antibacterial regimens that must be considered in clinic applications. Two essential criteria for the successful translation of a treatment method into clinical practice are prominent therapeutic efficacy and high biosafety. A significant challenge in the clinical application of natural products is their relatively low in vivo bioactivity, particularly when targeting colonic sites. This necessitates the use of suitable carriers for targeted drug delivery. While metal‐based materials have demonstrated antimicrobial activity, the potential toxicity of certain metal ions to the host must be carefully considered. Additionally, the application of PDT in CRC treatment is limited by restricted light penetration and potential phototoxicity. Therefore, exploring PSs in the NIR 2‐region and incorporating a built‐in light source may serve as effective measures to improve CRC treatment. Similarly, most AMPs exhibit short half‐lives and limited oral bioavailability due to their susceptibility to oxidation and enzymolysis and have yet to meet the clinical requirements of CRC treatment, especially oral administration. Ensuring stability and biodegradability are crucial considerations for polymers used in in vivo applications. Probiotics must withstand the acidic environment of the stomach and effectively target the CRC site after oral administration. To achieve antimicrobial efficacy in a clinical setting, it is essential to employ suitable carriers capable of delivering probiotics to the specific lesion site.
- *F. nucleatum* is the pathogen responsible for CRC. Therefore, it is crucial to simultaneously target both anticancer and antibacterial aspects during cancer treatment. Upon detecting the formation of CRC, the ideal therapeutic system should be designed.^[^ ^251^, ^252^ ^]^ First, the drug system must identify the receptor on the CRC cells and precisely target the cancer cells to prevent tumor spread. When the drug arrives at the CRC site, it is expected to precisely target a significant amount of pathogenic *F. nucleatum* in the intestinal environment and inhibit its growth. This concept allows the drug to combat CRC‐associated pathogenic bacteria while simultaneously treating CRC, thereby alleviating the burden of chemotherapy resistance and immunosuppression caused by *F. nucleatum* for subsequent treatment.
- Finally, much work has been done to showcase outstanding results in inhibiting *F. nucleatum* planktonic bacteria and disrupting the *F. nucleatum* biofilms, which provides different systems and strategies to combat the pathogenic bacteria of CRC in future.^[^ ^253^ ^]^ However, treating intracellular *F. nucleatum* infections poses a significant challenge. Following the invasion of tumor cells, bacteria augment the self‐protection mechanisms of tumor cells, thereby enhancing their survival in remote regions.^[^ ^254^ ^]^ Through our recent research, we have employed the oligomethyleneimine (OEI)‐LA‐phenylboronic acid (PBA) /Pt (IV) oxaliplatin prodrug (OXA‐COOH)‐modified PG (OLP/PP) system to effectively eliminate intratumoral *F. nucleatum* (both extracellular and intracellular) and inhibit tumor growth.^[^ ^255^ ^]^ The OLP/PP nanoassembly has been shown to have excellent biocompatibility through a series of experiments conducted in vitro and in vivo. Developing more approaches to eradicate intracellular *F. nucleatum* in the future is a new unexplored area that requires further investigation. This review summarized the strategies used to inhibit *F. nucleatum* in recent years, and numerous questions concerning *F. nucleatum* and CRC must be thoroughly investigated in the future.

## AUTHOR CONTRIBUTIONS

**Hongyu Liu**: Approach; research; data curation; original draft writing; visualization. **Yunjian Yu**: Editing; review; formal analysis. **Alideertu Dong**: Validation; review and editing. **Mahmoud Elsabahy**: Verification; review and editing. **Ying‐Wei Yang**: Project management; validation; data curation. **Hui Gao**: Ideas; materials; direction; finance.

## CONFLICT OF INTEREST

The authors declare no conflict of interest.
